# Preclinical characterization of INCB053914, a novel pan-PIM kinase inhibitor, alone and in combination with anticancer agents, in models of hematologic malignancies

**DOI:** 10.1371/journal.pone.0199108

**Published:** 2018-06-21

**Authors:** Holly Koblish, Yun-long Li, Niu Shin, Leslie Hall, Qian Wang, Kathy Wang, Maryanne Covington, Cindy Marando, Kevin Bowman, Jason Boer, Krista Burke, Richard Wynn, Alex Margulis, Gary W. Reuther, Que T. Lambert, Valerie Dostalik Roman, Ke Zhang, Hao Feng, Chu-Biao Xue, Sharon Diamond, Greg Hollis, Swamy Yeleswaram, Wenqing Yao, Reid Huber, Kris Vaddi, Peggy Scherle

**Affiliations:** 1 Incyte Corporation, Wilmington, Delaware, United States of America; 2 Department of Molecular Oncology, H. Lee Moffitt Cancer Center, Tampa, Florida, United States of America; Emory University, UNITED STATES

## Abstract

The Proviral Integration site of Moloney murine leukemia virus (PIM) serine/threonine protein kinases are overexpressed in many hematologic and solid tumor malignancies and play central roles in intracellular signaling networks important in tumorigenesis, including the Janus kinase–signal transducer and activator of transcription (JAK/STAT) and phosphatidylinositol 3-kinase (PI3K)/AKT pathways. The three PIM kinase isozymes (PIM1, PIM2, and PIM3) share similar downstream substrates with other key oncogenic kinases and have differing but mutually compensatory functions across tumors. This supports the therapeutic potential of pan-PIM kinase inhibitors, especially in combination with other anticancer agents chosen based on their role in overlapping signaling networks. Reported here is a preclinical characterization of INCB053914, a novel, potent, and selective adenosine triphosphate-competitive pan-PIM kinase inhibitor. *In vitro*, INCB053914 inhibited proliferation and the phosphorylation of downstream substrates in cell lines from multiple hematologic malignancies. Effects were confirmed in primary bone marrow blasts from patients with acute myeloid leukemia treated *ex vivo* and in blood samples from patients receiving INCB053914 in an ongoing phase 1 dose-escalation study. *In vivo*, single-agent INCB053914 inhibited Bcl-2–associated death promoter protein phosphorylation and dose-dependently inhibited tumor growth in acute myeloid leukemia and multiple myeloma xenografts. Additive or synergistic inhibition of tumor growth was observed when INCB053914 was combined with selective PI3Kδ inhibition, selective JAK1 or JAK1/2 inhibition, or cytarabine. Based on these data, pan-PIM kinase inhibitors, including INCB053914, may have therapeutic utility in hematologic malignancies when combined with other inhibitors of oncogenic kinases or standard chemotherapeutics.

## Introduction

The Proviral Integration site of Moloney murine leukemia virus (PIM) family of intracellular serine/threonine kinases consists of three highly homologous isozymes (PIM1, PIM2, and PIM3), each of which has been implicated in many hematologic and solid tumor malignancies [1]. PIM isozyme expression is elevated by cytokines and growth factors (eg, interleukin-6, interleukin-10, and granulocyte-macrophage colony-stimulating factor) that mediate signaling networks, which are themselves dysregulated in a wide range of cancers [1, 2]. For example, many of these cytokines signal through the Janus kinase (JAK and signal transducer and activator of transcription (STAT) pathway, which is both critical for normal hematopoiesis and associated with myeloproliferative neoplasms (MPNs) [3].

The PIM kinases are constitutively active and transcriptionally regulated by STAT1, STAT3, STAT5A, and STAT5B, which, in addition to tyrosine phosphorylation by JAK, are regulated by upstream serine-threonine kinases including extracellular signal-regulated kinases 1/2 [4], cyclin-dependent kinase 8 [5], p21-activated kinase 1 [6], and focal adhesion kinase (FAK) [6]. Of note, PIM kinase expression is regulated via a negative feedback loop involving phosphorylation of suppressors of cytokine signaling (SOCS) 1 and SOCS3 with subsequent inhibition of STAT5 [7, 8]. PIM kinases phosphorylate downstream substrates that are regulators of apoptosis, proliferation, migration, and cellular metabolism [1, 2]. Signaling networks mediated by PIM kinases share common nodes with other networks important for cell survival and proliferation [1, 2]. For example, Bcl-2–associated death promoter protein (BAD), 4E-BP1, and p70S6K are downstream substrates for both PIM kinases and phosphatidylinositol 3-kinase (PI3K) isozymes, including PI3Kδ, resulting in crosstalk between PIM kinase and PI3K/AKT/mammalian target of rapamycin complex 1 (mTORC1) signaling [1, 2, 9]. Notably, attenuation of PI3K/AKT/mTORC1 signaling via inhibition of AKT has been demonstrated to upregulate PIM1 expression [10].

The PIM kinase isozymes are expressed across cancers, but are primarily seen at elevated levels in hematologic malignancies. Importantly, the isozymes are differentially expressed; therefore, different isozymes or combinations thereof are active in different histologies [11–13]. For example, expression of both PIM1 and PIM2 is elevated in diffuse large B-cell lymphoma (DLBCL), whereas PIM2 alone is more highly expressed in B-cell chronic lymphocytic leukemia, acute myeloid leukemia (AML), and multiple myeloma (MM) [1, 12, 14–17]. Contrasting with PIM1 and PIM2, PIM3 is ubiquitously and uniformly expressed across hematologic malignancies, and is also more highly expressed in solid tumors (eg, pancreatic, gastric, and colon cancers) [1, 12]. PIM3 is also expressed in endothelial cells, where it colocalizes with FAK and is central in cell spreading and migration, suggesting a potential role in angiogenesis [18]. PIM1 overexpression is associated with a poor prognosis in various hematologic cancers and solid tumors (eg, mantle cell lymphoma [MCL], triple-negative breast, colorectal, pancreatic, gastroesophageal, and head and neck cancers) [19–26], PIM2 overexpression is associated with an aggressive clinical course in patients with DLBCL [27], and PIM3 overexpression is a risk factor for prostate cancer [28]. Of note, PIM kinase overexpression confers resistance to traditional chemotherapeutic agents and radiation therapy across several tumor types, including AML and MPNs [15, 25, 29–32].

Based on these observations, the PIM kinases have been proposed to be therapeutic targets in a variety of cancers [2]. The PIM isozymes have overlapping functions that are mutually compensatory, such that genetic or pharmacologic inhibition of one isozyme can lead to upregulation of one or both of the other two isozymes [33, 34]. Given this and the differential expression of individual PIM isozymes across tumors [11, 12], pan-PIM isozyme inhibition would appear to be most desirable for optimal efficacy [33, 35–38]. However, many of the pharmacologic PIM kinase adenosine triphosphate (ATP)-competitive inhibitors that have appeared in the literature thus far have been rationally designed to target PIM1 [39, 40]; PIM2 has displayed reduced susceptibility to these PIM1 inhibitors owing to secondary structure differences in the ATP-binding pocket [41]. Mice deficient in all three PIM kinases exhibit phenotypic abnormalities, including smaller overall size at maturity, decreased cellular responses to cytokines, and impaired hematopoiesis [42]. Of note, the phenotypic effects of PIM kinase deletion on hematopoiesis are similar to those observed upon STAT5 gene deletion [43], and to the dominant-negative effects of the expression of inactive, N-terminal truncated forms of STAT5 in mice [44–46]. Although these abnormalities are not trivial, mice in which *PIM1*, *PIM2*, and *PIM3* gene expression has been ablated remain viable and fertile, unlike phenotypes resulting from other gene deletions, which are typically embryonically lethal (eg, *MET*, *BRD4*, *FGFR1*, *JAK2*, *LSD1*, *MDM2*) [47–52]. Taken together, this suggests that pharmacologic pan-PIM isozyme inhibition as a therapeutic paradigm might result in relatively fewer concomitant toxicities.

**Fig 1:**
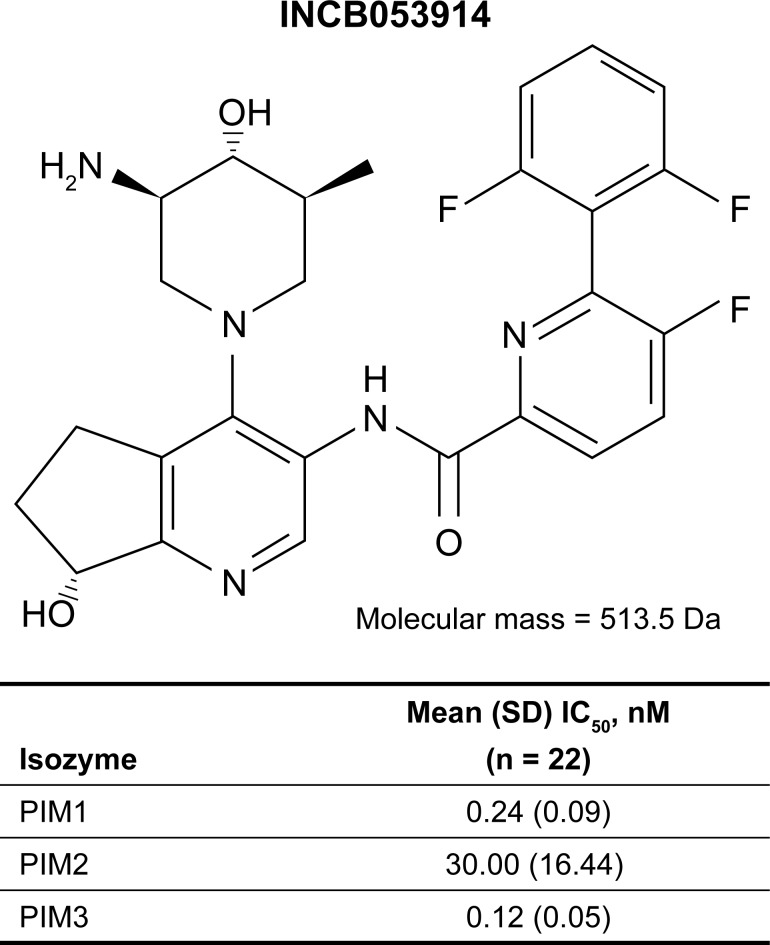
Structure of INCB053914 and IC_50_ values for the inhibition of PIM isozymes by INCB053914 in biochemical assays.

The substantial interconnectivity between signaling networks mediated by PIM kinases and those targeted by other anticancer agents raises the potential for combined additive or synergistic therapeutic effects. We hypothesized that pan-PIM isozyme inhibition alone or in combination with other anticancer agents might provide therapeutic benefit in patients with hematologic malignancies or solid tumors. A rational medicinal chemistry approach was undertaken to yield a compound that inhibits all three PIM isozymes, inhibits phosphorylation of downstream substrates both *in vitro* and *in vivo*, displays broad antiproliferative activity in multiple histologies, inhibits tumor growth, and meets pharmacokinetic and safety criteria to enable safe dosing in humans. Reported here is the first chemical disclosure and initial preclinical characterization of INCB053914 (Fig 1) [53], a novel, ATP-competitive, small-molecule, pan-inhibitor of PIM isozymes, alone or as part of combination regimens that include other anticancer agents chosen based on overlapping signaling networks.

## Materials and methods

INCB053914 (*N*-((*R*)-4-((3*R*,4*R*,5*S*)-3-amino-4-hydroxy-5-methylpiperidin-1-yl)-7-hydroxy-6,7-dihydro-5*H*-cyclopenta[*b*]pyridin-3-yl)-6-(2,6-difluorophenyl)-5-fluoropicolinamide phosphate), INCB050465, itacitinib (formerly INCB039110), and ruxolitinib (formerly INCB018424) were synthesized at Incyte Corporation (Wilmington, Delaware). Cytarabine was from Hanna Pharmaceutical Supply (Wilmington, Delaware). All tumor cell lines (MM, n = 11; AML, n = 15; B-cell malignancies, n = 13 [DLBCL, n = 9; MCL, n = 4]; T-cell acute lymphoblastic leukemia [T-ALL], n = 4; Hodgkin lymphoma, n = 1) were from ATCC^®^ (Rockville, Maryland) or DSMZ (Braunschweig, Germany), except for INA-6 (MM) cells, which were kindly provided by Dr. R. Burger (University Hospital Schleswig-Holstein, Kiel, Germany). Cultures were maintained according to supplier recommendations. Data plots and statistics were generated using GraphPad Prism version 5.0 (GraphPad Software, La Jolla, California).

### Biochemical PIM kinase activity

The activity of INCB053914 against the PIM1 and PIM3 kinases was measured using AlphaScreen^®^ assays (PerkinElmer, Inc., Shelton, Connecticut), and against PIM2 kinase using a time-resolved fluorescence resonance energy transfer assay (see Supporting Information, S1 File)

### Cell-based activity of INCB053914

The antiproliferative effects of INCB053914 on hematologic tumor cells were investigated using assays described in the Supporting Information (S2 File).

Inhibition of PIM kinase substrate phosphorylation (BAD, S6, p70S6K, and 4E-BP1) and the upregulation of PIM2 (chosen because of its reduced susceptibility to *in vitro* kinase inhibition) by INCB053914 was determined by incubating 10^6^ MOLM-16 (AML), Pfeiffer (DLBCL), KMS-12-PE (MM), and KMS-12-BM (MM) cells with INCB053914 at concentrations ranging from 0 (phosphate-buffered saline [PBS]) to 1 μM for 2 hours in Roswell Park Memorial Institute (RPMI) medium. Cells were centrifuged at 1,000 rpm for 10 minutes and lysed with 1× lysis buffer (Cell Signaling Technology, Danvers, Massachusetts) supplemented with 1 mM phenylmethane sulfonyl fluoride (Sigma-Aldrich, St Louis, Missouri) and proteinase inhibitor cocktail (CalBiochem, San Diego, California). Cell lysates were stored at –80°C before determining phosphoprotein and PIM2 levels by Western blotting using anti-pBAD (S112), anti-p4E-BP1 (S65), pp70S6K (T389), pS6 (S285/S286), and anti-PIM2 antibodies (Cell Signaling Technology).

The effect of INCB053914 on the level of pBAD was further investigated in KMS-12-BM and MOLM-16 cells, which were suspended in RPMI + 10% fetal bovine serum (FBS) and seeded into 96-well v-bottom polypropylene plates (Greiner, Munroe, North Carolina; 4 × 10^5^ cells/well/100 μl) in the presence of 5 μl INCB053914 at a final concentration range of 0.1 nM to 1,000 nM. After 2.5 hours at 37°C and 5% CO_2_, the cells were lysed in 100 μl of cell extraction buffer (Cell Signaling Technology) containing phenylmethane sulfonyl fluoride, Halt^TM^ phosphatase, and protease inhibitors (Thermo Fisher Scientific, Waltham, Massachusetts; Calbiochem). The concentration of pBAD protein in the cell lysates was quantified using a Human pBAD S112 ELISA Kit (Cell Signaling Technology).

The effects of INCB053914 on pBAD, pp70S6K, and p4E-BP1 levels and on the expression of PIM isozymes also were assessed *ex vivo* in primary bone marrow blasts (Stem Cell and Xenograft Core, University of Pennsylvania, Philadelphia, Pennsylvania) or in peripheral blood mononuclear cells (PBMCs) derived from whole blood samples obtained with informed written consent from adult patients with AML enrolled in an ongoing phase 1/2 dose-escalation trial, which was conducted in accordance with the study protocol approved by the respective institutional review boards or independent ethics committees. Only samples initially containing >90% viable cells were used (assessed by Trypan Blue staining). Immediately after thawing, the blasts were cultured in RPMI + 10% FBS with INCB053914 for 2 hours at 37°C, before lysis. For determinations of PIM2 expression in PBMCs, whole blood samples were treated overnight with increasing concentrations of INCB053914; total PBMCs were isolated by Ficoll-Paque density gradient centrifugation before lysis. The levels of PIM isozymes and phosphoproteins in cell lysates were detected by Western blotting using the same primary antibodies as above.

Erythroid colony formation assays were performed using primary cultures obtained from peripheral blood samples from patients with JAK2 V216F MPNs, as previously described [54]. Samples were obtained with informed written consent from adult patients through the Moffitt Cancer Center Total Cancer Care protocol (MCC 14690/ Liberty IRB #12.11.0023) approved by the Moffitt Cancer Center Scientific Review Committee.

### *In vivo* studies

Non- GLP studies intended to characterize the pharmacology of INCB053914 were conducted in accordance with Incyte Corporation's Animal Use Protocols and DuPont Stine-Haskell SOPs. Animals were housed in barrier facilities fully accredited by the Association for Assessment and Accreditation of Laboratory Animal Care, International. All of the procedures were conducted under the supervision of a veterinarian and in accordance with the U.S. Public Health Service Policy on Humane Care and Use of Laboratory Animals. In accordance with the study protocol approved by the Haskell Animal Welfare Committee (Protocol: HAWC008), animals were humanely euthanized (by carbon dioxide exposure) if they showed severe signs of pain or distress, if there was evidence of tumor necrosis or ulceration, if tumor growth impeded movement, if tumor weight exceeded 10% of body weight for 2 consecutive measurements, or if body weight loss exceeded 20% of baseline values. Analgesics and anesthetics could be used to minimize animal suffering and distress; neither were required during these studies. Animals on study were monitored twice daily and there were no unexpected deaths.

Female immune compromised (severe combined immunodeficiency [SCID]) mice (5–9 weeks of age; Charles River Laboratories, Wilmington, Massachusetts) were inoculated with MOLM-16, KG-1, or KMS-12-BM cells. A cell suspension in Dulbecco’s phosphate-buffered saline (DPBS) (1 × 10^8^ cells/ml) was mixed 1:1 (v/v) with matrigel, and 200 μl was injected subcutaneously into the flank of each mouse. Studies of INCB053914 combination regimens used xenograft models derived from INA-6 (MM) cells or Pfeiffer (DLBCL) cells. INA-6 tumor fragments were injected subcutaneously as brei (100 μl in Hank’s balanced salt solution [HBSS, 1:10 v/v]) into the flank of each mouse; Pfeiffer tumor fragments (1 cm × 1 cm) were directly implanted. The treatment of tumor-bearing mice started 7 to 24 days after tumor inoculation for efficacy studies and approximately 14 to 27 days after tumor inoculation for pharmacodynamic studies.

For efficacy studies, mice were sorted to obtain approximately equivalent mean tumor volumes in each group. The starting mean tumor volume in efficacy studies for all groups ranged from 158 mm^3^ to 249 mm^3^, and groups consisted of eight or nine animals. The mean tumor volume in pharmacodynamic studies for all groups ranged from 114 mm^3^ to 833 mm^3^, and groups consisted of three to eight animals. INCB053914 was administered by oral gavage twice a day for 7 to 19 days (suspension in 5% dimethylacetamide with 0.5% w/v methylcellulose [Sigma-Aldrich]) or by subcutaneous continuous infusion (Alzet osmotic pumps; flow rate 0.5 μl/h for efficacy studies and 1.0 μl/h for pharmacodynamic studies).

### INCB053914 single-agent therapy in xenograft models

Human MOLM-16 (AML) and KMS-12-BM (MM) xenografts were established in SCID mice. For the evaluation of intratumoral pBAD inhibition, tumor-bearing mice (three to eight mice/group) were treated with a single oral dose of INCB053914 at doses ranging from 0 (vehicle) to 100 mg/kg. In one representative experiment, mice were sacrificed 4 hours after a single dose, and tumors were collected to analyze the levels of pBAD relative to those of vehicle-administered controls. Plasma samples were collected for pharmacokinetic analysis.

For the evaluation of the effects of INCB053914 on tumor growth, tumor-bearing mice (MOLM-16 xenograft, eight mice/group; KMS-12-BM xenograft, nine mice/group) were dosed orally twice a day with INCB053914 at doses ranging from 0 (vehicle) to 100 mg/kg for 15 days.

Tumor volume was calculated in two dimensions using the equation: volume = [length × (width^2^)] / 2, where the larger number was length and the smaller number was width. Effects on tumor growth were reported as percent tumor growth inhibition calculated as (1 − [treatment volume / control volume]) × 100, where control volume was the vehicle/untreated tumor volume on a given day and treatment volume was any treatment group tumor volume on that same day. Statistical significance of differences between treatment and vehicle controls was assessed using analysis of variance single factor test.

### INCB053914 combination therapy in xenograft models

The PI3Kδ inhibitor INCB050465 (10 mg/kg once a day orally) was evaluated in combination with INCB053914 (30 mg/kg once a day orally) in mice bearing Pfeiffer (DLBCL) xenografts, a model sensitive to PI3Kδ inhibition (eight mice/group). Cytarabine was administered via intraperitoneal injection at a standard dose of 20 mg/kg twice a week, in combination with oral INCB053914 at a submaximally efficacious dose of 20 mg/kg twice a day for 12 days in mice bearing human KG-1 AML xenografts, a cytarabine-sensitive model (eight mice/group). The oral JAK1-selective inhibitor itacitinib (60 mg/kg twice a day orally) was assessed in combination with INCB053914 (100 mg/kg twice a day orally) after 8 days of administration to SCID mice bearing INA-6 (MM) xenografts (eight mice/group), a model sensitive to JAK inhibition [55].

Synergistic effects of INCB053914 in combination with INCB050465, cytarabine, or itacitinib, were assessed based on the Bliss independence model [56, 57]. In this model, the expected additive effect, with the assumption that concentration dependent effects of 2 drugs (A and B) are independent, is given by the equation *E_IND_ = E_A_ + E_B_ – E_A_ •E_B_*, where 0 ≤ *E*_*IND*_ ≤ 1; *E*_*A*_ and *E*_*B*_ are the magnitudes of effects of A and B alone at given concentrations.

The interaction between effects of A and B can be described by the equation *Combination Index* = *E*_*IND*_/ *E*_*OBS*_, where *E*_*OBS*_ is the observed combined effect of A + B expressed as a probability (0 ≤ *E*_*OBS*_ ≤ 1). If the point estimate of *Combination Index* and its upper 97.5% confidence limit are <1 then the combined effect is synergistic; if the 2-sided 95% confidence intervals (CI) encompass 1 then the combined effect is additive (ie, independent); if the point estimate of *Combination Index* and the lower 2.5% confidence limit are >1 then the combined effect of A + B are antagonistic.

Combined effects of INCB053914 with those of other anticancer agents on cell viability were assessed as described in Supporting Information.

### Bioanalysis of INCB053914 in mouse plasma

Concentrations of INCB053914 in plasma were analyzed using liquid chromatography tandem–mass spectrometry at 2, 4, 8, and 16 hours postdose (see Supporting Information, S3 File).

## Results

### Biochemical characterization of INCB053914

*In vitro* assays demonstrated that INCB053914 potently inhibits the activities of all three PIM isozymes with half maximal inhibitory concentration (IC_50_) values in the order of PIM1 ≈ PIM3 < PIM2 (Fig 1). Because of the reduced inhibitory potency towards PIM2 versus PIM1 and PIM3, PIM2 was chosen as the benchmark isozyme to ensure target coverage in subsequent experiments. INCB053914 was shown to be highly selective against a panel of more than 50 kinases (>475-fold selectivity; see Supporting Information S1 Table), except for RSK2 for which INCB053914 had modest potency (IC_50_ = 7.1 μM). Subsequent profiling against a broader panel of 192 kinases (PerkinElmer, Akron, Ohio; results not shown) confirmed this high selectivity; no kinase other than Per-Arnt-Sim (PAS) kinase was significantly inhibited by INCB053914 (100 nM). Further evaluation showed that INCB053914 had approximately equipotent effects on PIM2 and PAS kinase activities. Taken together, these results indicate that INCB053914 is a potent and highly specific pan-PIM inhibitor.

### INCB053914 inhibits tumor cell proliferation

**Fig 2:**
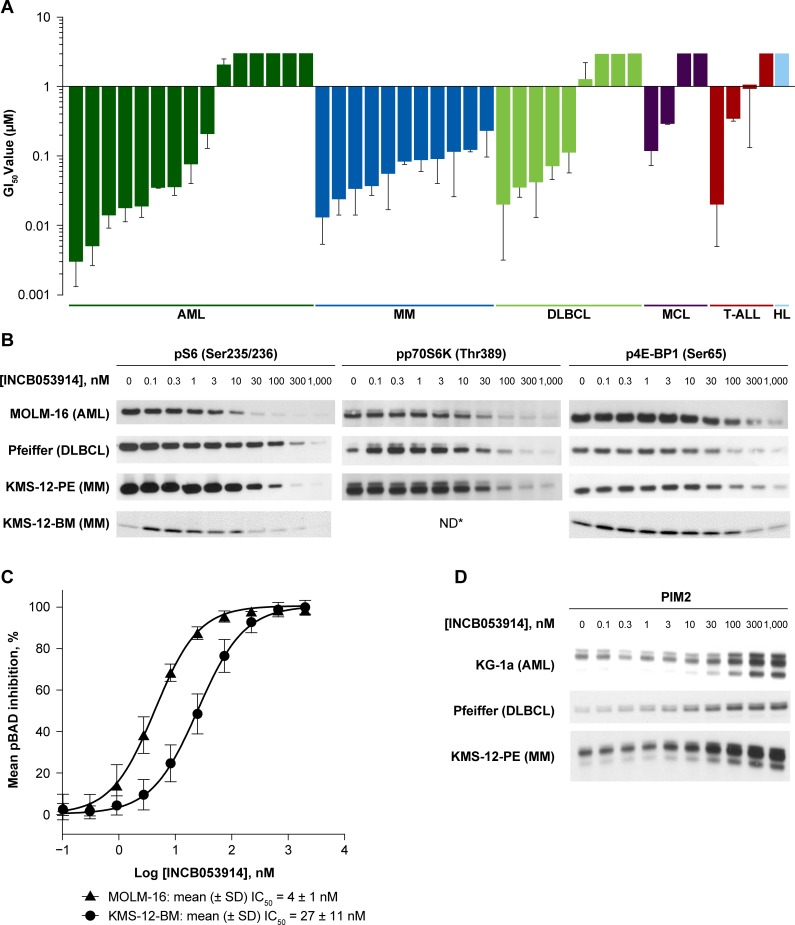
**INCB053914 inhibits cellular proliferation in hematologic tumor cell lines (A), inhibits phosphorylation of PIM substrates (B), including pBAD (C), and increases PIM2 expression (D) in hematologic tumor cell lines.** For all Western blots, actin controls confirmed equivalent loading. IC_50_ values for pBAD inhibition were determined by fitting the percent inhibition versus the log [INCB053914] data to sigmoidal dose–response (variable slope) curve. Error bars represent standard deviation. GI_50_ values >3 μM are not shown. *pP70S6K band intensities in KMS-12-BM (MM) cells were below the limit of detection at all INCB053914 concentrations tested. HL, Hodgkin lymphoma; ND, not determined. Original Western blot images are shown in Supporting Information S4 File.

The effects of INCB053914 on cellular proliferation were assessed in a panel of cell lines derived from hematologic malignancies including AML, MM, DLBCL, MCL, and T-ALL (Fig 2A). The mean growth inhibitory (GI_50_) values were <100 nM in 50% of cell lines tested (see Supporting Information S2 Table). Of note, INCB053914 inhibited proliferation in all MM cell lines tested, with mean GI_50_ values ranging from 13.2 nM to 230.0 nM. Mean GI_50_ values were <100 nM in eight of 15 (53%) AML cell lines tested; in particular, MOLM-16 and Kasumi-3 cells were extremely sensitive to inhibition by INCB053914 (mean GI_50_, 3.3 nM and 4.9 nM, respectively). Among DLBCL cell lines tested, the proliferation of Pfeiffer cells was the most sensitive to inhibition by INCB053914 (mean GI_50_, 19.5 nM). Taken together, these results show that INCB053914 has broad anti-proliferative activity against a variety of hematologic tumor cell lines. Based on their increased sensitivity to INCB053914MOLM-16, Pfeiffer, and KMS-12-PE/BM cell lines were selected for further study as models for AML, DLBCL, and MM, respectively.

### Decreased PIM kinase substrate phosphorylation and increased PIM2 expression as markers of INCB053914 activity

To confirm that the observed antiproliferative effects of INCB053914 in hematologic tumor cell lines were due to PIM inhibition, the effects of INCB053914 on PIM kinase-mediated signaling networks were investigated. Treatment with INCB053914 inhibited the phosphorylation of downstream PIM kinase substrates (p70S6K/S6 and 4E-BP1) in a dose-dependent manner in MOLM-16 (AML), Pfeiffer (DLBCL), and KMS-12-PE/BM (MM) cell lines (Fig 2B). PIM kinase-mediated phosphorylation of BAD in MOLM-16 and KMS-12-BM cells was particularly sensitive to inhibition by INCB053914 (mean IC_50_, 4 nM and 27 nM, respectively; Fig 2C). INCB053914 also increased PIM2 expression in KG-1a (AML), Pfeiffer, and KMS-12-PE cells (Fig 2D). The similar inhibitory potencies of the effects of INCB053914 on substrate phosphorylation and on proliferation in these cell lines provides evidence that PIM kinase inhibition is central to the antiproliferative effects of INCB053914.

**Fig 3:**
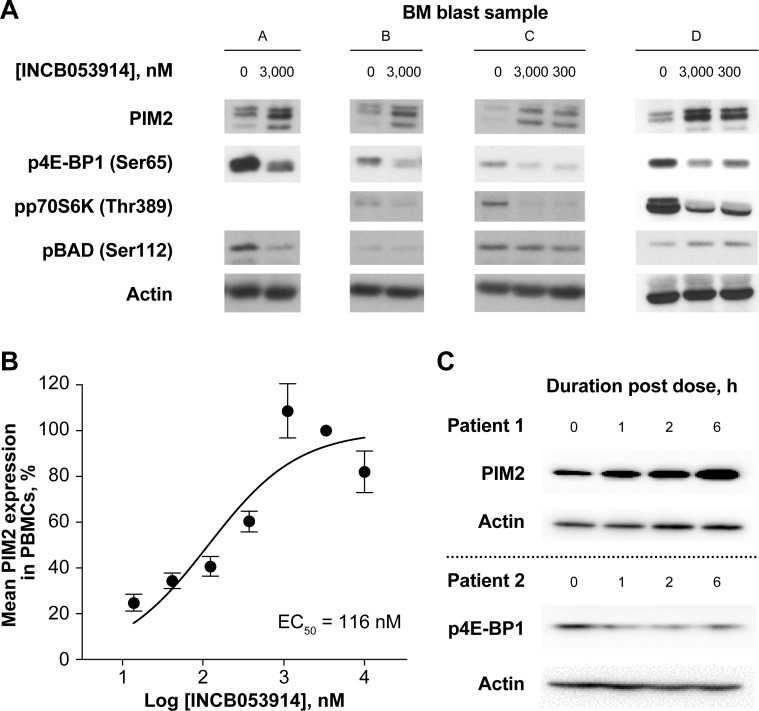
**INCB053914 inhibits phosphorylation of PIM substrates and increases PIM2 expression in primary bone marrow (BM) blasts (A), and increases PIM2 expression in PBMCs derived from whole blood samples from patients with AML (B). Pharmacodynamic effects of INCB053914 on PIM2 expression and 4E-BP1 phosphorylation in whole blood samples obtained 0 to 6 hours post-dose from two separate patients with AML enrolled in the ongoing phase 1/2 trial (C).** The EC_50_ for increased PIM2 expression were determined by fitting data to a sigmoidal dose–response (variable slope) curve. Original Western blot images are shown in Supporting Information S4 File.

*Ex vivo* treatment of primary bone marrow blasts from patients with AML with INCB053914 also decreased phosphorylation of p70S6K and 4E-BP1 and increased PIM2 expression (Fig 3A). Decreases in BAD phosphorylation were only seen in blasts from two of four patients. A concentration-dependent increase in PIM2 expression also was observed in PBMCs treated *ex vivo* with INCB053914 (Fig 3B). In addition, a pharmacodynamic assessment of 4 blood samples obtained from two separate patients with AML receiving INCB053914 100 mg twice a day in the ongoing phase 1/2 trial also demonstrated an increase in PIM2 expression levels and a decrease in 4E-BP1 phosphorylation (Fig 3C). These results indicate that inhibition of PIM kinases by INCB053914 elicits similar effects on downstream signaling in hematologic tumor cell lines to those in patient-derived primary blood cells.

### *Ex vivo* effects of INCB053914 on erythroid colony formation in cell cultures from patients with JAK2 V617F-positive MPNs

**Fig 4:**
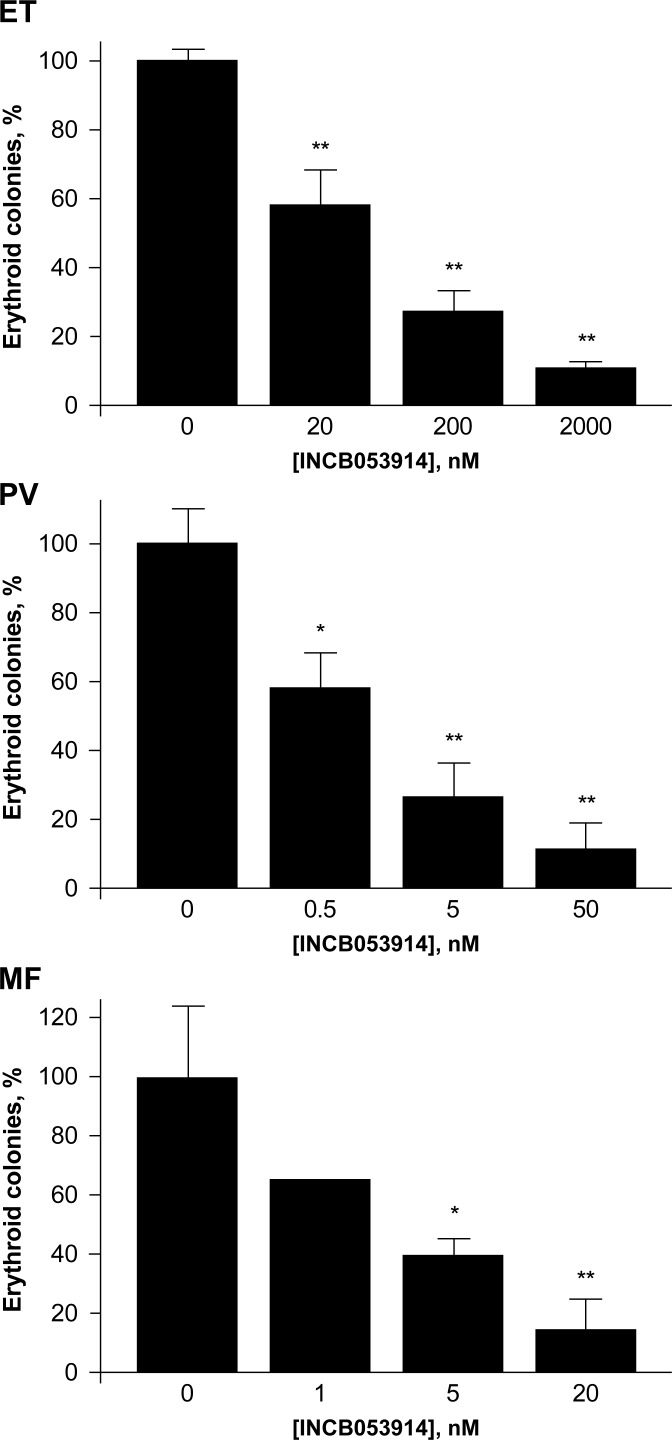
INCB053914 inhibits erythroid colony formation in patients with JAK2 V617F-positive MPNs. Error bars represent standard deviation. *p < 0.05; **p < 0.01.

PIM kinases are downstream mediators of JAK/STAT signaling, aberrant activation of which can lead to MPNs [3]. We therefore assessed the *ex vivo* effects of INCB053914 on neoplastic erythroid colony formation in primary cell cultures obtained from patients with polycythemia vera (PV), essential thrombocythemia (ET), and primary myelofibrosis (MF) who possess the activating JAK2 V617F mutation. In each case, a significant dose-dependent inhibition of colony formation was noted at INCB053914 concentrations as low as 0.5 nM (p < 0.05; Fig 4). These data indicate that downstream PIM kinase inhibition by INCB053914 blocks aberrant JAK/STAT activation, resulting in inhibited erythroid colony formation in patients with MPNs.

### INCB053914 inhibits PIM kinase-mediated signaling and tumor growth *in vivo*

**Fig 5:**
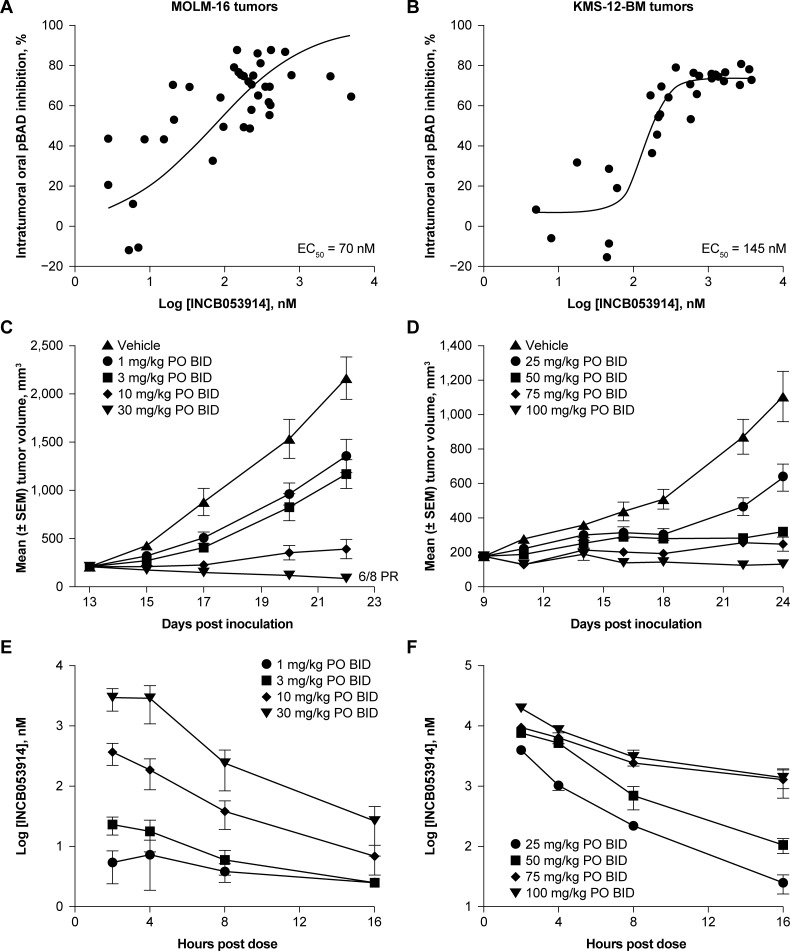
**INCB053914 inhibits the phosphorylation of BAD in mice bearing MOLM-16 (AML) (A) or KMS-12 (MM) tumors (B), and inhibits growth of MOLM-16 (AML) (C) and KMS-12 (MM) (D) tumors *in vivo*. Mean INCB053914 plasma concentrations were determined at 2, 4, 8, and 16 hours post oral administration in mice bearing MOLM-16 tumors (E) or KMS-12-BM tumors (F).** EC_50_ values for pBAD inhibition were determined by fitting data to a sigmoidal dose–response (variable slope) curve. Error bars represent standard error of the mean. BID, twice a day; PO, orally.

It was hypothesized that the inhibitory effects of INCB053914 on PIM kinase activities and cell proliferation *in vitro* would translate to inhibited PIM kinase-mediated signaling and tumor growth *in vivo*. Assessment of mice bearing MOLM-16 (AML) and KMS-12-BM (MM) tumors treated with INCB053914 demonstrated a dose-dependent inhibition of BAD phosphorylation relative to vehicle at 4 hours post dose (MOLM-16 tumors, IC_50_ = 70 nM; KMS-12-BM tumors, IC_50_ = 145 nM; Fig 5A and 5B). The potencies of these effects were similar to those observed for BAD phosphorylation in whole blood samples spiked with MOLM-16 or KMS-12-BM cells, when treated *ex vivo* with INCB053914 (MOLM-16 cells, IC_50_ = 76 nM; KMS-12-BM cells, IC_50_ = 134 nM).

Consistent with the observed pharmacodynamic effects, INCB053914 inhibited tumor growth in a dose-dependent manner in mice bearing MOLM-16 (AML) or KMS-12-BM (MM) (Fig 5C and 5D) tumors; in the MOLM-16 model, tumor growth inhibition was apparent at doses as low as 1 mg/kg. Maximal growth inhibition in MOLM-16 tumors (96%) was observed at 30 mg/kg twice a day where six of eight mice had a partial regression (PR; defined as a ≥50% decrease in tumor volume vs. that at treatment start). INCB053914-mediated KMS-12-BM tumor growth inhibition was 43%, 71%, 77%, and 88% at doses of 25, 50, 75, and 100 mg/kg twice a day, respectively. INCB053914 was tolerated at all doses tested. A pharmacokinetic analysis of INCB053914 plasma concentrations up to 16 hours post oral administration in MOLM-16 and KMS-12-BM tumor-bearing mice suggested dose proportionality (Fig 5E and 5F). Maximal antitumor activity was observed in these models when the trough plasma concentration of INCB053914 exceeded the IC_50_ for pBAD inhibition determined in whole blood samples spiked with MOLM-16 or KMS-12-BM cells.

### Synergistic effects of INCB053914 in combination with other anticancer agents

Because PIM kinases integrate signals from multiple intracellular signaling networks [1, 2], rational combinations of INCB053914 with anticancer agents that target other signaling pathways important in cell proliferation and survival were investigated.

#### Combination with PI3Kδ inhibition

**Fig 6:**
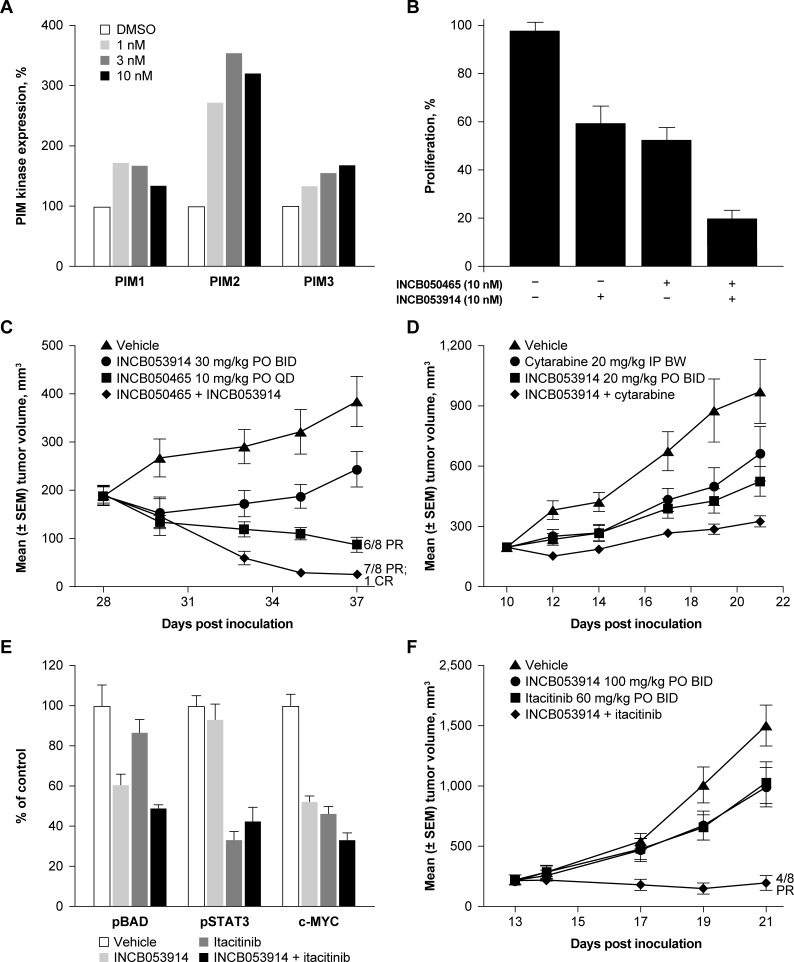
**Effects of the selective PI3Kδ inhibitor, INCB050465, on PIM isozyme expression in Pfeiffer DLBCL cells (A). Effects of INCB053914 alone, or in combination with INCB050465, on the *in vitro* proliferation of Pfeiffer DLBCL cells (B). Effects of INCB053914 alone or in combination with INCB050465 on tumor growth in a DLBCL xenograft model (C); with cytarabine on tumor growth in an AML xenograft model (D); with the JAK1-selective inhibitor, itacitinib, on BAD, STAT3 phosphorylation, and MYC levels (E), and on tumor growth in an INA-6 MM xenograft model (F).** Error bars represent standard error of the mean. BW, twice a week; CR, complete regression; IP, intraperitoneally; QD, once a day.

PIM kinases and PI3K/AKT/mTORC1 share similar downstream substrates, including BAD, 4E-BP1, and p70S6K, and both upregulate PIM2 expression [1, 2, 9, 10]. This suggests that inhibition of PIM kinases may modulate the interconnectivity between these two networks. Consistent with this, synergistic effects of PIM kinase inhibitors with pan-PI3K and PI3Kα selective inhibitors have been shown [10, 58–60]. We therefore assessed the effects of INCB053914 in combination with INCB050465, a highly selective and highly potent PI3Kδ inhibitor currently being assessed in phase 2 trials for the treatment of hematologic malignancies. In an *in vitro* assay, INCB050465 elicited concentration-dependent increases in the expression of all three PIM isozymes in Pfeiffer DLBCL cells relative to baseline, with PIM2 expression being increased the most (~2.5-fold; Fig 6A). The proliferation of Pfeiffer cells was inhibited by both INCB053914 and INCB050465 as single agents; the two agents combined had synergistic antiproliferative effects (point estimate of Bliss *Combination Index* [2-sided 95% CI] = 0.923 [0.845, 0.979]; Fig 6B). Notably, the combined administration of INCB053914 with INCB050465 in Pfeiffer cell xenografts resulted in synergistic tumor growth inhibition compared with either agent alone. Among eight treated mice receiving the combination, seven had a PR and one had a complete regression (defined as ≥50% and 100% decreases in tumor volume vs that at treatment start, respectively; Fig 6C).

#### Combination with cytarabine

Cytarabine induces the expression of CD25 via activation of STAT5 [61], which then leads to transcriptional activation of PIM kinases [62]. Therefore, the effects of INCB053914 on the antitumor activity of cytarabine were assessed in SCID mice bearing KG-1 (AML) tumors (Fig 6D). Tumor growth was inhibited by administration of either INCB053914 or cytarabine alone, and combined administration of INCB053914 with cytarabine resulted in additive inhibition (point estimate of Bliss *Combination Index* [2-sided 95% CI] = 0.967 [0.495, 1.205]; Fig 6D). All regimens were well tolerated.

#### Combination with JAK inhibition

Cytokines and growth factors elicit hematopoietic cell proliferation and differentiation via the JAK/STAT signaling pathway [63]. Cellular PIM kinase activity also is regulated by cytokines via JAK/STAT5 signaling at the level of transcription and translation [1, 2]. In addition, PIM2 in part regulates IL-6 gene expression [64], which is also induced by STAT5 [65]. Based on this, it has been hypothesized that PIM2 might potentiate STAT5 activation via a feed-forward mechanism leading to sustained STAT-mediated cellular proliferation [61]. Therefore, we investigated whether combined PIM kinase and JAK inhibition might have synergistic/additive antitumor effects. In mice bearing JAK-dependent INA-6 (MM) tumors, administration of INCB053914 or itacitinib as single-agents led to a decrease in the level of pBAD and pSTAT3, respectively, as expected. Administration of INCB053914 plus itacitinib in combination reduced pBAD and pSTAT3 levels to a similar extent as single-agent INCB053914 and single-agent itacitinib, respectively (Fig 6E). Notably, administration of either INCB053914 or itacitinib alone resulted in decreased MYC levels, with slightly stronger effects observed with the combination regimen. In a separate experiment, INCB053914 had negligible effects on pSTAT3(Y705) levels, and PIM kinase substrate phosphorylation was unaffected by ruxolitinib (JAK1/2 selective inhibitor), and itacitinib (JAK1 selective inhibitor; Supplementary Information S1 Fig). A synergistic inhibition of tumor growth was observed with INCB053914 plus itacitinib compared with either agent alone in INA-6 xenografts (agents were administered at doses lower than required for full efficacy; point estimate of Bliss *Combination Index* [2-sided 95% CI] = 0.720 [0.256, 0.924]; Fig 6F). Partial responses were seen in four of eight mice tested. In a separate experiment, similar effects on tumor growth were obtained for a combination of INCB053914 with ruxolitinib, showing that JAK1-selective and JAK1/2 dual inhibitors behave similarly in these models (data not shown).

In addition to the above rational combinations, the ability of INCB053914 to potentiate the inhibitory effects of a panel of 65 anticancer agents on the viability of MM cells (KMS-11, KMS-12-BM, MM1.S) also was assessed. Among these agents, 19 showed varying degrees of additivity/synergy with INCB053914 (Supplementary Information S2 Fig). Particularly potent were combinations of INCB053914 with PI3K/AKT pathway inhibitors, chemotherapeutic agents including azacitidine, and small-molecule inhibitors including ibrutinib, that have utility in treating patients with hematologic malignancies.

## Discussion

PIM kinases function in cells as integrators of several signaling networks that overlap with those that can be targeted by other anticancer agents and that share common downstream substrates [1, 2]. For example, signaling networks mediated by PIM kinases overlap substantially with the PI3K/AKT/mTORC1 pathway [1, 2, 9]; PIM kinases and mTORC1 both block the anti-apoptotic function of BAD [2]. In addition, both PIM2 and mTORC1 phosphorylate 4E-BP1 via parallel signaling pathways, which then upregulates cap-dependent protein translation [1, 17]. We therefore hypothesized that along with potential single-agent activity, there also could be synergy between the *in vitro* and *in vivo* antitumor effects of INCB053914 and those of other rationally chosen anticancer agents.

In this study, single-agent INCB053914 potently inhibited the activity of all three PIM kinase isozymes and displayed high selectivity for these kinases. Single-agent INCB053914 upregulated PIM2 and inhibited phosphorylation of multiple substrates in hematologic cancer cell lines as well as in patient-derived primary AML cells. These included the pro-apoptotic protein, BAD, in MM (KMS-12-BM) and AML (MOLM-16) cell lines, as well as other downstream signaling proteins important in cell survival, growth, and migration. In keeping with a common role of PIM kinases across hematologic malignancies, single-agent INCB053914 inhibited proliferation in a diverse panel of hematologic tumor cell lines, and inhibited tumor growth *in vivo* in both AML and MM xenograft models at tolerated doses.

Consistent with the interplay between PIM kinase- and PI3K/AKT/mTORC1-mediated signaling pathways, INCB053914 enhanced the inhibitory effects of INCB050465, a highly selective PI3Kδ inhibitor, on tumor growth in a xenograft model of DLBCL. Of note, inhibition of the AKT has been shown to upregulate the PIM1 [10], which may confer resistance to PI3K inhibitor therapy in breast cancer [66]. Consistent with this, it is shown here that INCB050465 increased expression of all three PIM isozymes. These data indicate that combining PIM kinase inhibition by INCB053914 and PI3Kδ inhibition by INCB050465 may provide a potential therapeutic approach for ameliorating this compensatory mechanism of resistance.

Cytarabine has been shown to upregulate the expression of CD25 via STAT5 activation in the AML cell line, KG-1 [61]. pSTAT5 and CD25 are negative prognostic biomarkers in AML [67–69], and the presence of CD25^+^ blasts has been suggested to be associated with chemotherapy relapse [67, 70, 71]. Notably, although PIM kinase expression is regulated by STAT5, PIM kinase inhibition suppressed CD25 expression, a surrogate of STAT5 activity, in AML cells [61]. Moreover, KG-1 (AML) cells selected for cytarabine resistance displayed increased sensitivity to PIM inhibition versus unselected cells [61]. Experiments performed previously in our laboratory also have demonstrated that among a panel of AML cell lines including KG-1 cells, those expressing CD25 were more sensitive to inhibition by INCB053914 [72]. In keeping with these observations, it is shown here that INCB053914 synergistically increased the inhibitory effects of cytarabine on tumor growth in KG-1 xenografts.

The observation that PIM kinases are downstream regulators of JAK/STAT signaling [2, 62] supports the potential utility of PIM kinase inhibition in combination with JAK inhibition. Thus, INCB053914 synergistically enhanced the inhibitory potency of the JAK1-selective inhibitor, itacitinib, and the JAK1/2-selective inhibitor, ruxolitinib, on tumor growth in xenograft models of MM. Of note, PIM kinase inhibition alone did not impact on STAT3 activity, based on the observation that pSTAT3(Y705) levels were unaffected by INCB053914; also JAK inhibition alone did not affect downstream PIM kinase substrate phosphorylation. The demonstrated activity of ruxolitinib in patients with MPNs also led us to assess the activity of INCB053914 in primary cultures from patients with JAK2 V617F-positive PV, ET, and MF. INCB053914 concentrations as low as 1 nM to 5 nM were effective at inhibiting the formation of neoplastic erythroid colonies. Further studies exploring the activity of INCB053914 in combination with ruxolitinib in additional samples suggest enhanced activity (G.W. Reuther, manuscript in preparation).

Although there have been several other PIM kinase-targeted agents described in the literature [33, 35–38], most are specific for PIM1 and few inhibit all three PIM isozymes. In particular, the development of an ATP-competitive inhibitor of PIM2 has been hindered by the relatively high binding affinity of PIM2 for ATP versus the other two isozymes [33]. INCB053914 potently inhibited the activities of all three PIM isozymes, which is important for avoiding compensatory activity and for maximizing therapeutic efficacy [33, 34, 73]. PIM447 and AZD1208 are also potent and selective pan-PIM inhibitors that show similar activity *in vitro* and *in vivo* to INCB053914 [35, 37, 54]. For example, as observed for INCB053914, AZD1208 displayed antiproliferative effects in several AML cell lines, which were relatively more potent for MOLM-16 and Kasumi-3 cells [74, 75]. The effects of AZD1208 were predominantly cytostatic versus apoptotic; similar to INCB053914, the growth inhibitory effects of AZD1208 reflected a dose-dependent inhibition of downstream mTOR signaling, including a reduction in mTOR, 4E-BP1, and p70S6K/S6 phosphorylation [74, 75]. PIM447 is currently being evaluated in combination with ruxolitinib and LEE011 for the treatment of patients with myelofibrosis in a phase 1 clinical trial (ClinicalTrials.gov identifier: NCT02370706).

In summary, the present data, obtained using *in vitro* and *in vivo* model systems, support the potential therapeutic benefit of targeting PIM kinase-mediated signaling networks, particularly when these overlap with networks mediated by other anticancer agents. Thus, consistent with a common role of PIM kinases across hematologic malignancies, single-agent INCB053914 inhibited constitutive PIM kinase-mediated signaling and proliferation in multiple hematologic tumor cell lines and in primary tumor cells and demonstrated notable inhibitory effects on tumor growth. These effects were additively or synergistically enhanced by coadministration with standard chemotherapy, including cytarabine, and targeted therapies, suggesting that INCB053914 may have particular utility in combination regimens. Moreover, the observation that among a panel of 65 anticancer agents, 19 showed additive/synergistic antitumor effects with INCB053914 indicates considerable latitude in tailoring combination regimens for the treatment of different cancers. Based on these results, a phase 1/2 dose-escalation trial is currently recruiting patients to assess the safety and preliminary efficacy of INCB053914 alone or combined with the PI3Kδ inhibitor INCB050465, azacitidine, intermediate-dose cytarabine, or ruxolitinib, for the treatment of advanced malignancies (ClinicalTrials.gov identifier: NCT02587598).

## Supporting information

S1 Fig Effects of INCB053914 on STAT3(Y705) and PIM kinase substrate phosphorylation in INA-6 cells treated for 24 hours in culture. (DOCX)

S2 Fig Synergistic effect of INCB053914 in combination with other anticancer agents against viability of MM cell lines. (DOCX)

S1 Table Selectivity of INCB053914 for PIM kinases compared with 56 kinases representative of the kinome [ATP] = 1 mM. (DOCX)

S2 Table Antiproliferative activity of INCB053914 in hematologic tumor cell lines. (DOCX)

S1 File In vitro PIM kinase activity. (DOCX)

S2 File Cell-based activity of INCB053914. (DOCX)

S3 File Bioanalysis of INCB053914 in mouse plasma. (DOCX)

S4 File Original Western blot images. (DOCX)

## References

[1] Nawijn MC, Alendar A, Berns A. For better or for worse: the role of Pim oncogenes in tumorigenesis. Nat Rev Cancer. 2011;11(1):23–34. Epub 2010/12/15. doi: 10.1038/nrc2986 . 21150935 10.1038/nrc2986

[2] Mondello P, Cuzzocrea S, Mian M. Pim kinases in hematological malignancies: where are we now and where are we going? J Hematol Oncol. 2014;7:95 Epub 2014/12/11. doi: 10.1186/s13045-014-0095-z ; PubMed Central PMCID: PMC4266197. 25491234 10.1186/s13045-014-0095-z PMC4266197

[3] Vainchenker W, Constantinescu SN. JAK/STAT signaling in hematological malignancies. Oncogene. 2013;32(21):2601–13. Epub 2012/08/08. doi: 10.1038/onc.2012.347 . 22869151 10.1038/onc.2012.347

[4] Pircher TJ, Petersen H, Gustafsson JA, Haldosen LA. Extracellular signal-regulated kinase (ERK) interacts with signal transducer and activator of transcription (STAT) 5a. Mol Endocrinol. 1999;13(4):555–65. Epub 1999/04/09. doi: 10.1210/mend.13.4.0263 . 10194762 10.1210/mend.13.4.0263

[5] Rzymski T, Mikula M, Zylkiewicz E, Dreas A, Wiklik K, Golas A, et al SEL120-34A is a novel CDK8 inhibitor active in AML cells with high levels of serine phosphorylation of STAT1 and STAT5 transactivation domains. Oncotarget. 2017;8(20):33779–95. Epub 2017/04/20. doi: 10.18632/oncotarget.16810 ; PubMed Central PMCID: PMC5464911. 28422713 10.18632/oncotarget.16810 PMC5464911

[6] Chatterjee A, Ghosh J, Ramdas B, Mali RS, Martin H, Kobayashi M, et al Regulation of Stat5 by FAK and PAK1 in Oncogenic FLT3- and KIT-Driven Leukemogenesis. Cell reports. 2014;9(4):1333–48. Epub 2014/12/03. doi: 10.1016/j.celrep.2014.10.039 ; PubMed Central PMCID: PMC4380442. 25456130 10.1016/j.celrep.2014.10.039 PMC4380442

[7] Paukku K, Silvennoinen O. STATs as critical mediators of signal transduction and transcription: lessons learned from STAT5. Cytokine & growth factor reviews. 2004;15(6):435–55. Epub 2004/11/25. doi: 10.1016/j.cytogfr.2004.09.001 . 15561601 10.1016/j.cytogfr.2004.09.001

[8] Peltola KJ, Paukku K, Aho TL, Ruuska M, Silvennoinen O, Koskinen PJ. Pim-1 kinase inhibits STAT5-dependent transcription via its interactions with SOCS1 and SOCS3. Blood. 2004;103(10):3744–50. Epub 2004/02/07. doi: 10.1182/blood-2003-09-3126 . 14764533 10.1182/blood-2003-09-3126

[9] Schatz JH, Oricchio E, Wolfe AL, Jiang M, Linkov I, Maragulia J, et al Targeting cap-dependent translation blocks converging survival signals by AKT and PIM kinases in lymphoma. J Exp Med. 2011;208(9):1799–807. Epub 2011/08/24. doi: 10.1084/jem.20110846 ; PubMed Central PMCID: PMC3171093. 21859846 10.1084/jem.20110846 PMC3171093

[10] Cen B, Mahajan S, Wang W, Kraft AS. Elevation of receptor tyrosine kinases by small molecule AKT inhibitors in prostate cancer is mediated by Pim-1. Cancer Res. 2013;73(11):3402–11. Epub 2013/04/16. doi: 10.1158/0008-5472.CAN-12-4619 ; PubMed Central PMCID: PMC3680595. 23585456 10.1158/0008-5472.CAN-12-4619 PMC3680595

[11] Shah N, Pang B, Yeoh KG, Thorn S, Chen CS, Lilly MB, et al Potential roles for the PIM1 kinase in human cancer–a molecular and therapeutic appraisal. Eur J Cancer. 2008;44(15):2144–51. Epub 2008/08/22. doi: 10.1016/j.ejca.2008.06.044 . 18715779 10.1016/j.ejca.2008.06.044

[12] Lu J, Zavorotinskaya T, Dai Y, Niu XH, Castillo J, Sim J, et al Pim2 is required for maintaining multiple myeloma cell growth through modulating TSC2 phosphorylation. Blood. 2013;122(9):1610–20. Epub 2013/07/03. doi: 10.1182/blood-2013-01-481457 ; PubMed Central PMCID: PMC3953014. 23818547 10.1182/blood-2013-01-481457 PMC3953014

[13] Akagi K, Li J, Broutian TR, Padilla-Nash H, Xiao W, Jiang B, et al Genome-wide analysis of HPV integration in human cancers reveals recurrent, focal genomic instability. Genome Res. 2014;24(2):185–99. Epub 2013/11/10. doi: 10.1101/gr.164806.113 ; PubMed Central PMCID: PMC3912410. 24201445 10.1101/gr.164806.113 PMC3912410

[14] Keane NA, Reidy M, Natoni A, Raab MS, O'Dwyer M. Targeting the Pim kinases in multiple myeloma. Blood Cancer J. 2015;5:e325 Epub 2015/07/18. doi: 10.1038/bcj.2015.46 ; PubMed Central PMCID: PMC4526774. 26186558 10.1038/bcj.2015.46 PMC4526774

[15] Chen LS, Redkar S, Bearss D, Wierda WG, Gandhi V. Pim kinase inhibitor, SGI-1776, induces apoptosis in chronic lymphocytic leukemia cells. Blood. 2009;114(19):4150–7. Epub 2009/09/08. doi: 10.1182/blood-2009-03-212852 ; PubMed Central PMCID: PMC2774551. 19734450 10.1182/blood-2009-03-212852 PMC2774551

[16] Cohen AM, Grinblat B, Bessler H, Kristt D, Kremer A, Schwartz A, et al Increased expression of the hPim-2 gene in human chronic lymphocytic leukemia and non-Hodgkin lymphoma. Leukemia & lymphoma. 2004;45(5):951–5. Epub 2004/08/05. doi: 10.1080/10428190310001641251 . 15291354 10.1080/10428190310001641251

[17] Tamburini J, Green AS, Bardet V, Chapuis N, Park S, Willems L, et al Protein synthesis is resistant to rapamycin and constitutes a promising therapeutic target in acute myeloid leukemia. Blood. 2009;114(8):1618–27. Epub 2009/05/22. doi: 10.1182/blood-2008-10-184515 . 19458359 10.1182/blood-2008-10-184515

[18] Zhang P, Wang H, Min X, Wang Y, Tang J, Cheng J, et al Pim-3 is expressed in endothelial cells and promotes vascular tube formation. Journal of cellular physiology. 2009;220(1):82–90. Epub 2009/02/21. doi: 10.1002/jcp.21733 . 19229879 10.1002/jcp.21733

[19] Peng YH, Li JJ, Xie FW, Chen JF, Yu YH, Ouyang XN, et al Expression of pim-1 in tumors, tumor stroma and tumor-adjacent mucosa co-determines the prognosis of colon cancer patients. PLoS ONE. 2013;8(10):e76693 Epub 2013/10/12. doi: 10.1371/journal.pone.0076693 ; PubMed Central PMCID: PMC3792018. 24116137 10.1371/journal.pone.0076693 PMC3792018

[20] Xu J, Xiong G, Cao Z, Huang H, Wang T, You L, et al PIM-1 contributes to the malignancy of pancreatic cancer and displays diagnostic and prognostic value. J Exp Clin Cancer Res. 2016;35(1):133 Epub 2016/09/07. doi: 10.1186/s13046-016-0406-z ; PubMed Central PMCID: PMC5011911. 27596051 10.1186/s13046-016-0406-z PMC5011911

[21] Zhou Z, Zhang R, Wang R, Zhang Y, Xu L, Chen J, et al Expression of Pim-3 in colorectal cancer and its relationship with prognosis. Tumour Biol. 2016;37(7):9151–6. Epub 2016/01/16. doi: 10.1007/s13277-016-4806-7 . 26768612 10.1007/s13277-016-4806-7

[22] Hsi ED, Jung SH, Lai R, Johnson JL, Cook JR, Jones D, et al Ki67 and PIM1 expression predict outcome in mantle cell lymphoma treated with high dose therapy, stem cell transplantation and rituximab: a Cancer and Leukemia Group B 59909 correlative science study. Leuk Lymphoma. 2008;49(11):2081–90. Epub 2008/11/21. doi: 10.1080/10428190802419640 ; PubMed Central PMCID: PMC4011712. 19021050 10.1080/10428190802419640 PMC4011712

[23] Liu HT, Wang N, Wang X, Li SL. Overexpression of Pim-1 is associated with poor prognosis in patients with esophageal squamous cell carcinoma. J Surg Oncol. 2010;102(6):683–8. Epub 2010/06/15. doi: 10.1002/jso.21627 . 20544717 10.1002/jso.21627

[24] Warnecke-Eberz U, Bollschweiler E, Drebber U, Metzger R, Baldus SE, Hölscher AH, et al Prognostic impact of protein overexpression of the proto-oncogene PIM-1 in gastric cancer. Anticancer Res. 2009;29(11):4451–5. Epub 2009/12/25. . 20032391

[25] Peltola K, Hollmen M, Maula SM, Rainio E, Ristamäki R, Luukkaa M, et al Pim-1 kinase expression predicts radiation response in squamocellular carcinoma of head and neck and is under the control of epidermal growth factor receptor. Neoplasia. 2009;11(7):629–36. Epub 2009/07/02. ; PubMed Central PMCID: PMC2697349. 19568408 10.1593/neo.81038 PMC2697349

[26] Horiuchi D, Camarda R, Zhou AY, Yau C, Momcilovic O, Balakrishnan S, et al PIM1 kinase inhibition as a targeted therapy against triple-negative breast tumors with elevated MYC expression. Nat Med. 2016;22(11):1321–9. Epub 2016/11/01. doi: 10.1038/nm.4213 ; PubMed Central PMCID: PMC5341692. 27775705 10.1038/nm.4213 PMC5341692

[27] Gómez-Abad C, Pisonero H, Blanco-Aparicio C, Roncador G, González-Menchén A, Martinez-Climent JA, et al PIM2 inhibition as a rational therapeutic approach in B-cell lymphoma. Blood. 2011;118(20):5517–27. Epub 2011/09/23. doi: 10.1182/blood-2011-03-344374 . 21937691 10.1182/blood-2011-03-344374

[28] Qu Y, Zhang C, Du E, Wang A, Yang Y, Guo J, et al Pim-3 is a critical risk factor in development and prognosis of prostate cancer. Med Sci Monit. 2016;22:4254–60. Epub 2016/11/09. doi: 10.12659/MSM.898223 ; PubMed Central PMCID: PMC5108370. 27826135 10.12659/MSM.898223 PMC5108370

[29] Huang SM, Wang A, Greco R, Li Z, Barberis C, Tabart M, et al Combination of PIM and JAK2 inhibitors synergistically suppresses MPN cell proliferation and overcomes drug resistance. Oncotarget. 2014;5(10):3362–74. Epub 2014/05/17. doi: 10.18632/oncotarget.1951 ; PubMed Central PMCID: PMC4102815. 24830942 10.18632/oncotarget.1951 PMC4102815

[30] Kelly KR, Espitia CM, Taverna P, Choy G, Padmanabhan S, Nawrocki ST, et al Targeting PIM kinase activity significantly augments the efficacy of cytarabine. Br J Haematol. 2012;156(1):129–32. Epub 2011/06/22. doi: 10.1111/j.1365-2141.2011.08792.x . 21689092 10.1111/j.1365-2141.2011.08792.x

[31] Isaac M, Siu A, Jongstra J. The oncogenic PIM kinase family regulates drug resistance through multiple mechanisms. Drug Resist Updat. 2011;14(4–5):203–11. Epub 2011/05/24. doi: 10.1016/j.drup.2011.04.002 . 21601509 10.1016/j.drup.2011.04.002

[32] Musiani D, Hammond DE, Cirillo L, Erriquez J, Olivero M, Clague MJ, et al PIM2 kinase is induced by cisplatin in ovarian cancer cells and limits drug efficacy. J Proteome Res. 2014;13(11):4970–82. Epub 2014/08/08. doi: 10.1021/pr500651n . 25099161 10.1021/pr500651n

[33] Garcia PD, Langowski JL, Wang Y, Chen M, Castillo J, Fanton C, et al Pan-PIM kinase inhibition provides a novel therapy for treating hematologic cancers. Clin Cancer Res. 2014;20(7):1834–45. Epub 2014/01/30. doi: 10.1158/1078-0432.CCR-13-2062 . 24474669 10.1158/1078-0432.CCR-13-2062

[34] van der Lugt NM, Domen J, Verhoeven E, Linders K, van der Gulden H, Allen J, et al Proviral tagging in E mu-myc transgenic mice lacking the Pim-1 proto-oncogene leads to compensatory activation of Pim-2. EMBO J. 1995;14(11):2536–44. Epub 1995/06/01. ; PubMed Central PMCID: PMC398367. 7781606 10.1002/j.1460-2075.1995.tb07251.x PMC398367

[35] Paíno T, Garcia-Gomez A, González-Méndez L, San-Segundo L, Hernández-García S, López-Iglesias AA, et al The novel pan-PIM kinase inhibitor, PIM447, displays dual antimyeloma and bone-protective effects, and potently synergizes with current standards of care. Clin Cancer Res. 2017;23(1):225–38. Epub 2016/07/22. doi: 10.1158/1078-0432.CCR-16-0230 . 27440267 10.1158/1078-0432.CCR-16-0230

[36] Cervantes-Gomez F, Lavergne B, Keating MJ, Wierda WG, Gandhi V. Combination of Pim kinase inhibitors and Bcl-2 antagonists in chronic lymphocytic leukemia cells. Leuk Lymphoma. 2015:[Epub 28 September 2015]. doi: 10.3109/10428194.2015.1063141 Epub 2015/06/20. ; PubMed Central PMCID: PMC4814351. 26088877 10.3109/10428194.2015.1063141 PMC4814351

[37] Harada M, Benito J, Yamamoto S, Kaur S, Arslan D, Ramirez S, et al The novel combination of dual mTOR inhibitor AZD2014 and pan-PIM inhibitor AZD1208 inhibits growth in acute myeloid leukemia via HSF pathway suppression. Oncotarget. 2015;6(35):37930–47. Epub 2015/10/17. doi: 10.18632/oncotarget.6122 ; PubMed Central PMCID: PMC4741975. 26473447 10.18632/oncotarget.6122 PMC4741975

[38] Meja K, Stengel C, Sellar R, Huszar D, Davies BR, Gale RE, et al PIM and AKT kinase inhibitors show synergistic cytotoxicity in acute myeloid leukaemia that is associated with convergence on mTOR and MCL1 pathways. Br J Haematol. 2014;167(1):69–79. Epub 2014/07/01. doi: 10.1111/bjh.13013 . 24975213 10.1111/bjh.13013

[39] Merkel AL, Meggers E, Ocker M. PIM1 kinase as a target for cancer therapy. Expert opinion on investigational drugs. 2012;21(4):425–36. Epub 2012/03/06. doi: 10.1517/13543784.2012.668527 . 22385334 10.1517/13543784.2012.668527

[40] Arunesh GM, Shanthi E, Krishna MH, Sooriya Kumar J, Viswanadhan VN. Small molecule inhibitors of PIM1 kinase: July 2009 to February 2013 patent update. Expert opinion on therapeutic patents. 2014;24(1):5–17. Epub 2013/10/18. doi: 10.1517/13543776.2014.848196 . 24131033 10.1517/13543776.2014.848196

[41] Bullock AN, Russo S, Amos A, Pagano N, Bregman H, Debreczeni JE, et al Crystal structure of the PIM2 kinase in complex with an organoruthenium inhibitor. PLoS ONE. 2009;4(10):e7112 Epub 2009/10/21. doi: 10.1371/journal.pone.0007112 ; PubMed Central PMCID: PMC2743286. 19841674 10.1371/journal.pone.0007112 PMC2743286

[42] Mikkers H, Nawijn M, Allen J, Brouwers C, Verhoeven E, Jonkers J, et al Mice deficient for all PIM kinases display reduced body size and impaired responses to hematopoietic growth factors. Mol Cell Biol. 2004;24(13):6104–15. doi: 10.1128/MCB.24.13.6104-6115.2004 15199164 10.1128/MCB.24.13.6104-6115.2004 PMC480904

[43] Teglund S, McKay C, Schuetz E, van Deursen JM, Stravopodis D, Wang D, et al Stat5a and Stat5b proteins have essential and nonessential, or redundant, roles in cytokine responses. Cell. 1998;93(5):841–50. Epub 1998/06/18. . 9630227 10.1016/s0092-8674(00)81444-0

[44] Moriggl R, Gouilleux-Gruart V, Jahne R, Berchtold S, Gartmann C, Liu X, et al Deletion of the carboxyl-terminal transactivation domain of MGF-Stat5 results in sustained DNA binding and a dominant negative phenotype. Molecular and cellular biology. 1996;16(10):5691–700. Epub 1996/10/01. ; PubMed Central PMCID: PMC231569. 8816482 10.1128/mcb.16.10.5691 PMC231569

[45] Li G, Wang Z, Zhang Y, Kang Z, Haviernikova E, Cui Y, et al STAT5 requires the N-domain to maintain hematopoietic stem cell repopulating function and appropriate lymphoid-myeloid lineage output. Experimental hematology. 2007;35(11):1684–94. Epub 2007/11/03. doi: 10.1016/j.exphem.2007.08.026 ; PubMed Central PMCID: PMC2134320. 17976521 10.1016/j.exphem.2007.08.026 PMC2134320

[46] Li G, Miskimen KL, Wang Z, Xie XY, Brenzovich J, Ryan JJ, et al STAT5 requires the N-domain for suppression of miR15/16, induction of bcl-2, and survival signaling in myeloproliferative disease. Blood. 2010;115(7):1416–24. Epub 2009/12/17. doi: 10.1182/blood-2009-07-234963 ; PubMed Central PMCID: PMC2826763. 20008792 10.1182/blood-2009-07-234963 PMC2826763

[47] Parganas E, Wang D, Stravopodis D, Topham DJ, Marine JC, Teglund S, et al Jak2 is essential for signaling through a variety of cytokine receptors. Cell. 1998;93(3):385–95. Epub 1998/05/20. . 9590173 10.1016/s0092-8674(00)81167-8

[48] Houzelstein D, Bullock SL, Lynch DE, Grigorieva EF, Wilson VA, Beddington RS. Growth and early postimplantation defects in mice deficient for the bromodomain-containing protein Brd4. Molecular and cellular biology. 2002;22(11):3794–802. Epub 2002/05/09. doi: 10.1128/MCB.22.11.3794-3802.2002 ; PubMed Central PMCID: PMC133820. 11997514 10.1128/MCB.22.11.3794-3802.2002 PMC133820

[49] Deng CX, Wynshaw-Boris A, Shen MM, Daugherty C, Ornitz DM, Leder P. Murine FGFR-1 is required for early postimplantation growth and axial organization. Genes & development. 1994;8(24):3045–57. Epub 1994/12/15. . 8001823 10.1101/gad.8.24.3045

[50] Wang J, Scully K, Zhu X, Cai L, Zhang J, Prefontaine GG, et al Opposing LSD1 complexes function in developmental gene activation and repression programmes. Nature. 2007;446(7138):882–7. Epub 2007/03/30. doi: 10.1038/nature05671 . 17392792 10.1038/nature05671

[51] Huh CG, Factor VM, Sanchez A, Uchida K, Conner EA, Thorgeirsson SS. Hepatocyte growth factor/c-met signaling pathway is required for efficient liver regeneration and repair. Proceedings of the National Academy of Sciences of the United States of America. 2004;101(13):4477–82. Epub 2004/04/09. doi: 10.1073/pnas.0306068101 ; PubMed Central PMCID: PMC384772. 15070743 10.1073/pnas.0306068101 PMC384772

[52] Jones SN, Roe AE, Donehower LA, Bradley A. Rescue of embryonic lethality in Mdm2-deficient mice by absence of p53. Nature. 1995;378(6553):206–8. Epub 1995/11/09. doi: 10.1038/378206a0 . 7477327 10.1038/378206a0

[53] Vechorkin O, Feng H, Li Y-L, Mei S, Wang A, Zhu W, et al., inventors; Incyte Corporation, assignee. Pyridineamine compounds useful as Pim kinase inhibitors. United States patent US 9,540,347 B2. 2016.

[54] Mazzacurati L, Lambert QT, Pradhan A, Griner LN, Huszar D, Reuther GW. The PIM inhibitor AZD1208 synergizes with ruxolitinib to induce apoptosis of ruxolitinib sensitive and resistant JAK2-V617F-driven cells and inhibit colony formation of primary MPN cells. Oncotarget. 2015;6(37):40141–57. Epub 2015/10/17. doi: 10.18632/oncotarget.5653 ; PubMed Central PMCID: PMC4741885. 26472029 10.18632/oncotarget.5653 PMC4741885

[55] Li J, Favata M, Kelley JA, Caulder E, Thomas B, Wen X, et al INCB16562, a JAK1/2 selective inhibitor, is efficacious against multiple myeloma cells and reverses the protective effects of cytokine and stromal cell support. Neoplasia. 2010;12(1):28–38. Epub 2010/01/15. ; PubMed Central PMCID: PMC2805881. 20072651 10.1593/neo.91192 PMC2805881

[56] Foucquier J, Guedj M. Analysis of drug combinations: current methodological landscape. Pharmacol Res Perspect. 2015;3(3):e00149 Epub 2015/07/15. doi: 10.1002/prp2.149 ; PubMed Central PMCID: PMC4492765. 26171228 10.1002/prp2.149 PMC4492765

[57] Petraitis V, Petraitiene R, Hope WW, Meletiadis J, Mickiene D, Hughes JE, et al Combination therapy in treatment of experimental pulmonary aspergillosis: in vitro and in vivo correlations of the concentration- and dose- dependent interactions between anidulafungin and voriconazole by Bliss independence drug interaction analysis. Antimicrobial agents and chemotherapy. 2009;53(6):2382–91. Epub 2009/03/25. doi: 10.1128/AAC.00329-09 ; PubMed Central PMCID: PMC2687223. 19307368 10.1128/AAC.00329-09 PMC2687223

[58] Blanco-Aparicio C, Collazo AM, Oyarzabal J, Leal JF, Albarán MI, Lima FR, et al Pim 1 kinase inhibitor ETP-45299 suppresses cellular proliferation and synergizes with PI3K inhibition. Cancer Lett. 2011;300(2):145–53. Epub 2010/11/06. doi: 10.1016/j.canlet.2010.09.016 . 21051136 10.1016/j.canlet.2010.09.016

[59] Chen K, Yang J, Li J, Wang X, Chen Y, Huang S, et al eIF4B is a convergent target and critical effector of oncogenic Pim and PI3K/Akt/mTOR signaling pathways in Abl transformants. Oncotarget. 2016;7(9):10073–89. Epub 2016/02/06. doi: 10.18632/oncotarget.7164 ; PubMed Central PMCID: PMC4891105. 26848623 10.18632/oncotarget.7164 PMC4891105

[60] Shin N, Koblish H, Covington M, Li Y, Wang K, Wang Q, et al INCB050465, a novel PI3Kδ inhibitor, synergizes with PIM protein kinase inhibition to cause tumor regression in a model of DLBCL [abstract]. Cancer Res. 2015;75(15 Suppl):2671 doi: 10.1158/1538-7445.am2015-2671

[61] Guo Z, Wang A, Zhang W, Levit M, Gao Q, Barberis C, et al PIM inhibitors target CD25-positive AML cells through concomitant suppression of STAT5 activation and degradation of MYC oncogene. Blood. 2014;124(11):1777–89. Epub 2014/07/10. doi: 10.1182/blood-2014-01-551234 . 25006129 10.1182/blood-2014-01-551234

[62] Gozgit JM, Bebernitz G, Patil P, Ye M, Parmentier J, Wu J, et al Effects of the JAK2 inhibitor, AZ960, on Pim/BAD/BCL-xL survival signaling in the human JAK2 V617F cell line SET-2. J Biol Chem. 2008;283(47):32334–43. Epub 2008/09/09. doi: 10.1074/jbc.M803813200 . 18775810 10.1074/jbc.M803813200

[63] Silvennoinen O, Saharinen P, Paukku K, Takaluoma K, Kovanen P. Cytokine receptor signal transduction through Jak tyrosine kinases and Stat transcription factors. APMIS. 1997;105(7):497–509. Epub 1997/07/01. . 9269296 10.1111/j.1699-0463.1997.tb05047.x

[64] Yang J, Li X, Hanidu A, Htut TM, Sellati R, Wang L, et al Proviral integration site 2 is required for interleukin-6 expression induced by interleukin-1, tumour necrosis factor-alpha and lipopolysaccharide. Immunology. 2010;131(2):174–82. Epub 2010/05/15. doi: 10.1111/j.1365-2567.2010.03286.x ; PubMed Central PMCID: PMC2967263. 20465571 10.1111/j.1365-2567.2010.03286.x PMC2967263

[65] Kawashima T, Murata K, Akira S, Tonozuka Y, Minoshima Y, Feng S, et al STAT5 induces macrophage differentiation of M1 leukemia cells through activation of IL-6 production mediated by NF-kappaB p65. J Immunol. 2001;167(7):3652–60. Epub 2001/09/21. . 11564778 10.4049/jimmunol.167.7.3652

[66] Le X, Antony R, Razavi P, Treacy DJ, Luo F, Ghandi M, et al Systematic functional characterization of resistance to PI3K inhibition in breast cancer. Cancer Discov. 2016;6(10):1134–47. Epub 2016/09/09. doi: 10.1158/2159-8290.CD-16-0305 ; PubMed Central PMCID: PMC5050154. 27604488 10.1158/2159-8290.CD-16-0305 PMC5050154

[67] Gönen M, Sun Z, Figueroa ME, Patel JP, Abdel-Wahab O, Racevskis J, et al CD25 expression status improves prognostic risk classification in AML independent of established biomarkers: ECOG phase 3 trial, E1900. Blood. 2012;120(11):2297–306. Epub 2012/08/03. doi: 10.1182/blood-2012-02-414425 ; PubMed Central PMCID: PMC3447784. 22855599 10.1182/blood-2012-02-414425 PMC3447784

[68] Fujiwara SI, Muroi K, Yamamoto C, Hatano K, Okazuka K, Sato K, et al CD25 as an adverse prognostic factor in elderly patients with acute myeloid leukemia. Hematology. 2017;22(6):347–53. Epub 2017/01/18. doi: 10.1080/10245332.2016.1276240 . 28097942 10.1080/10245332.2016.1276240

[69] Brady A, Gibson S, Rybicki L, Hsi E, Saunthararajah Y, Sekeres MA, et al Expression of phosphorylated signal transducer and activator of transcription 5 is associated with an increased risk of death in acute myeloid leukemia. European journal of haematology. 2012;89(4):288–93. Epub 2012/06/26. doi: 10.1111/j.1600-0609.2012.01825.x . 22725130 10.1111/j.1600-0609.2012.01825.x

[70] Terwijn M, Feller N, van Rhenen A, Kelder A, Westra G, Zweegman S, et al Interleukin-2 receptor alpha-chain (CD25) expression on leukaemic blasts is predictive for outcome and level of residual disease in AML. Eur J Cancer. 2009;45(9):1692–9. Epub 2009/03/27. doi: 10.1016/j.ejca.2009.02.021 . 19321337 10.1016/j.ejca.2009.02.021

[71] Cerny J, Yu H, Ramanathan M, Raffel GD, Walsh WV, Fortier N, et al Expression of CD25 independently predicts early treatment failure of acute myeloid leukaemia (AML). Br J Haematol. 2013;160(2):262–6. Epub 2012/11/03. doi: 10.1111/bjh.12109 . 23116454 10.1111/bjh.12109

[72] Koblish H, Shin N, Hall L, O'Connor S, Wang Q, Wang K, et al Activity of the pan-PIM kinase inhibitor INCB053914 in models of acute myelogenous leukemia [abstract]. Cancer Res. 2015;75(15 Suppl):5416 doi: 10.1158/1538-7445.am2015-5416

[73] Meeker TC, Nagarajan L, ar-Rushdi A, Croce CM. Cloning and characterization of the human PIM-1 gene: a putative oncogene related to the protein kinases. J Cell Biochem. 1987;35(2):105–12. Epub 1987/10/01. doi: 10.1002/jcb.240350204 . 3429489 10.1002/jcb.240350204

[74] Keeton EK, McEachern K, Dillman KS, Palakurthi S, Cao Y, Grondine MR, et al AZD1208, a potent and selective pan-Pim kinase inhibitor, demonstrates efficacy in preclinical models of acute myeloid leukemia. Blood. 2014;123(6):905–13. Epub 2013/12/24. doi: 10.1182/blood-2013-04-495366 ; PubMed Central PMCID: PMC3916880. 24363397 10.1182/blood-2013-04-495366 PMC3916880

[75] Chen LS, Yang JY, Liang H, Cortes JE, Gandhi V. Protein profiling identifies mTOR pathway modulation and cytostatic effects of Pim kinase inhibitor, AZD1208, in acute myeloid leukemia. Leuk Lymphoma. 2016;57(12):2863–73. Epub 2016/04/08. doi: 10.3109/10428194.2016.1166489 . 27054578 10.3109/10428194.2016.1166489 PMC5650058

