# Supplementary material for: Preclinical characterization of INCB053914, a novel pan-PIM kinase inhibitor, alone and in combination with anticancer agents, in models of hematologic malignancies
Source: PLoS One. 2018 Jun 21;13(6):e0199108. doi: 10.1371/journal.pone.0199108 (PMC6013247; doi:10.1371/journal.pone.0199108)
Supplement: S1 File — (DOCX) [file pone.0199108.s005.docx]

**Supporting Information (S1 File)**

**Preclinical characterization of INCB053914, a novel pan-PIM kinase inhibitor, alone and in combination with anticancer agents, in models of hematologic malignancies**

Holly Koblish, Yun-long Li, Niu Shin, Leslie Hall, Qian Wang, Kathy Wang, Maryanne Covington, Cindy Marando, Kevin Bowman, Jason Boer, Krista Burke, Richard Wynn, Alex Margulis, Gary W. Reuther, Que T. Lambert, Valerie Dostalik Roman, Ke Zhang, Hao Feng, Chu-Biao Xue, Sharon Diamond, Greg Hollis, Swamy Yeleswaram, Wenqing Yao, Reid Huber, Kris Vaddi, Peggy Scherle

**Methods**

*In vitro* PIM kinase activity

***AlphaScreen assay.*** Reactions (20 μl) were carried out in white 384-well polystyrene plates (Greiner Bio-One, Kremsmünster, Austria) dotted with 0.8 μl of the compound in dimethylsulfoxide in the assay buffer (50 mM Tris, pH 7.5, 0.01% Tween-20, 5 mM MgCl_2_, 0.01% bovine serum albumin [BSA], 5 mM dithiothreitol) containing 0.05 μM Biotin-labeled Bcl-2–associated death promoter protein peptide substrate (AnaSpec, Fremont, California),
1 mM ATP, and 2.5 pM PIM1 (Invitrogen, Thermo Fisher Scientific, Waltham, Massachusetts), or 1.25 pM PIM3 (Millipore, Billerica, Massachusetts) enzyme. After 1 hour at 25°C, the reactions were stopped with 10 μl STOP Buffer (150 mM Tris, pH 7.5, 150 mM NaCl, 75 mM ethylenediaminetetraacetic acid [EDTA], 0.01% Tween‑20, 0.3% BSA) supplemented with Phospho-Bad (Ser112) antibody (Cell Signaling Technology, Danvers, Massachusetts) diluted 666-fold, and Streptavidin donor beads (PerkinElmer, Waltham, Massachusetts), along with Protein‑A acceptor beads (PerkinElmer, Akron, Ohio), at 15 μg/ml each. Supplementation of STOP buffer with beads and stopping the reactions were carried out under reduced light. Prior to stopping the reactions, the STOP buffer was preincubated with the beads for 1 hour in the dark at room temperature. After the reactions were stopped, the plates were incubated for 1 hour in the dark at room temperature and then read on a PHERAstar FS plate reader (BMG Labtech, Cary, North Carolina) under reduced light.

***Time-resolved fluorescence resonance energy transfer assay.*** Reactions (20 μl) were carried out in white 384-well polystyrene plates dotted with 0.8 μl of the compound in dimethylsulfoxide in the assay buffer (50 mM Tris, pH 7.5, 0.01% Tween-20, 5 mM MgCl_2_, 0.01% BSA, 5 mM dithiothreitol) containing 0.05 μM fluorescein-labeled CREBtide peptide substrate (Invitrogen), 1 mM ATP, and 1 nM PIM2 (Invitrogen). After 2 hours at 25°C, reactions were stopped with 10 μl RT-FRET dilution buffer (Invitrogen) supplemented with 30 mM EDTA and 1.5 nM LanthaScreen Tb-CREB pSer133 antibody (Invitrogen). After a 30-minute incubation at room temperature, plates were read on a PHERAstar FS plate reader (BMG Labtech).
