# Supplementary material for: Preclinical characterization of INCB053914, a novel pan-PIM kinase inhibitor, alone and in combination with anticancer agents, in models of hematologic malignancies
Source: PLoS One. 2018 Jun 21;13(6):e0199108. doi: 10.1371/journal.pone.0199108 (PMC6013247; doi:10.1371/journal.pone.0199108)
Supplement: S2 File — (DOCX) [file pone.0199108.s006.docx]

**Supporting Information (S2 File)**

**Preclinical characterization of INCB053914, a novel pan-PIM kinase inhibitor, alone and in combination with anticancer agents, in models of hematologic malignancies**

Holly Koblish, Yun-long Li, Niu Shin, Leslie Hall, Qian Wang, Kathy Wang, Maryanne Covington, Cindy Marando, Kevin Bowman, Jason Boer, Krista Burke, Richard Wynn, Alex Margulis, Gary W. Reuther, Que T. Lambert, Valerie Dostalik Roman, Ke Zhang, Hao Feng, Chu-Biao Xue, Sharon Diamond, Greg Hollis, Swamy Yeleswaram, Wenqing Yao, Reid Huber, Kris Vaddi, Peggy Scherle

**Methods**

Cell-based activity of INCB053914

***Cell proliferation assays.*** Cells were plated in their respective culture medium into 96-well ultralow binding plates (Corning Inc., Corning, New York) (2 × 10^3^ cells/200 μl/well), and INCB053914 was added to achieve a final concentration range of 0 to 1 nM. After 3 to 4 days, [^3^H]-thymidine (1 μCi/well) (PerkinElmer) in PBS (10 μl) was added to the cell culture for an additional 12 hours. The incorporated radioactivity was separated by filtration with water through GF/B filters (Packard Bioscience, Meriden, Connecticut) and measured by liquid scintillation counting with a TopCount (Packard Bioscience).

***In vitro combination screen***. Compounds for studies of the combined effects of INCB053914 with those of other anticancer agents on cell viability were purchased from Selleckchem (Houston, TX) and prepared as 5 mM stock solutions in DMSO (Sigma-Aldrich, St. Louis, MO). These selected compounds were anticancer agents with reasonably well known mechanism of action and most of them are approved drugs or already in clinical trials. Multiple myeloma cell line KMS-11 was purchased from JCRB (Osaka, Japan). Multiple myeloma cell lines KMS-12-BM and MM1.S were purchased from DSMZ (Braunschweig, Germany) and ATCC (Manassas, VA), respectively. All of the cell lines were maintained in RPMI-1640 medium (Life Technologies, Grand Island, NY) supplemented with 10% fetal bovine serum (HyClone/Thermo Fisher Scientific, Logan, UT) in a humidified 5% carbon dioxide atmosphere at 37°C. Cells were seeded in 384-well tissue culture plates (Greiner Bio-One, catalog 781946, Monroe, NC) in 25 μl medium at an initial seeding density of 400 cells/well for all the cell lines. For the IC50 determination with 11-point dose–response curves, 5 ul of serially diluted compound in media were added to each well. Each well contained 30 μl cells in media with final DMSO of 0.2%. Cells were incubated at 37°C in humidified air with 5% CO_2_.Cell viability was assessed using the CellTiter-Glo® assay (Promega, Madison, WI). One-hundred-and-twenty hours after dosing, the test plates were allowed to stand at room temperature for 10 min before adding 30 μl of CellTiter-Glo® reagent (Promega, Madison, WI). After 15 min of incubation at room temperature, luminescence was measured on TopCount (Perkin Elmer, Waltham, MA). Luminescence data were converted to growth fraction by normalization to the DMSO control. IC_50_ determination was performed by fitting the curve of percent control activity versus the log of the compound concentration using the GraphPad Prism 6.0 software. Synergy was assessed based on an average combination index calculated according to Chou and Talaly (Chou TC, Talaly P. J Biol Chem. 1977;252:6438-6442).
