# Supplementary material for: Preclinical characterization of INCB053914, a novel pan-PIM kinase inhibitor, alone and in combination with anticancer agents, in models of hematologic malignancies
Source: PLoS One. 2018 Jun 21;13(6):e0199108. doi: 10.1371/journal.pone.0199108 (PMC6013247; doi:10.1371/journal.pone.0199108)
Supplement: S3 File — (DOCX) [file pone.0199108.s007.docx]

**Supporting Information (S3 File)**

**Preclinical characterization of INCB053914, a novel pan-PIM kinase inhibitor, alone and in combination with anticancer agents, in models of hematologic malignancies**

Holly Koblish, Yun-long Li, Niu Shin, Leslie Hall, Qian Wang, Kathy Wang, Maryanne Covington, Cindy Marando, Kevin Bowman, Jason Boer, Krista Burke, Richard Wynn, Alex Margulis, Gary W. Reuther, Que T. Lambert, Valerie Dostalik Roman, Ke Zhang, Hao Feng, Chu-Biao Xue, Sharon Diamond, Greg Hollis, Swamy Yeleswaram, Wenqing Yao, Reid Huber, Kris Vaddi, Peggy Scherle

**Methods**

Bioanalysis of INCB053914 in mouse plasma

Blood samples from each study were collected using EDTA and centrifuged to obtain plasma. The calibration curves were prepared in mouse plasma (BioreclamationIVT, Westbury, New York). Samples (50 µl) were placed in a 2-ml square well plate (Costar, Corning, New York). Acetonitrile/methanol 9/1 v/v (550 µl) containing an internal standard was added for precipitation of the protein. The 96-well plate was covered, vortexed for 5 minutes, and centrifuged for 10 minutes at 3,000 rpm. Supernatant (400 µl) was transferred to a 1-ml round well plate (Costar). The samples were dried under nitrogen for 30 minutes (Biotage SPE Dry, Charlotte, North Carolina) and then reconstituted in 200 µl of acetonitrile/water 1/9 v/v. The samples were analyzed for INCB053914 by liquid chromatography tandem–mass spectrometry using gradient high-performance liquid chromatography and atmospheric pressure chemical ionization.
