# Supplementary material for: Preclinical characterization of INCB053914, a novel pan-PIM kinase inhibitor, alone and in combination with anticancer agents, in models of hematologic malignancies
Source: PLoS One. 2018 Jun 21;13(6):e0199108. doi: 10.1371/journal.pone.0199108 (PMC6013247; doi:10.1371/journal.pone.0199108)

**Supporting Information (S4 file)**

**Preclinical characterization of INCB053914, a novel pan-PIM kinase inhibitor, alone and in combination with anticancer agents, in models of hematologic malignancies**

Holly Koblish, Yun-long Li, Niu Shin, Leslie Hall, Qian Wang, Kathy Wang, Maryanne Covington, Cindy Marando, Kevin Bowman, Jason Boer, Krista Burke, Richard Wynn, Alex Margulis, Gary W. Reuther, Que T. Lambert, Valerie Dostalik Roman, Ke Zhang, Hao Feng, Chu-Biao Xue, Sharon Diamond, Greg Hollis, Swamy Yeleswaram, Wenqing Yao, Reid Huber, Kris Vaddi, Peggy Scherle

**Original Western Blot Images**

**Figure 2 Panel B**

**MOLM-16 (AML) - pS6 (Ser235/236)**

**
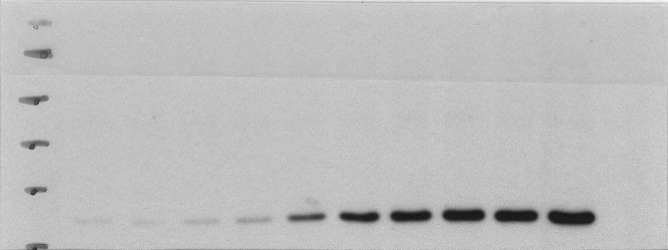
**

**MOLM-16 (AML) – pp70S6K (Thr389)**

**
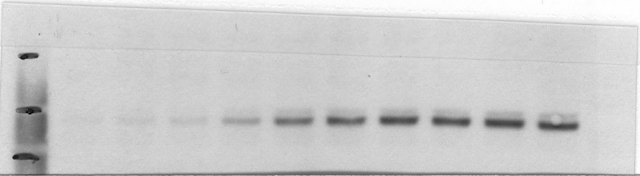
**

**MOLM-16 (AML) – p4E-BP1 (Ser65)**

**
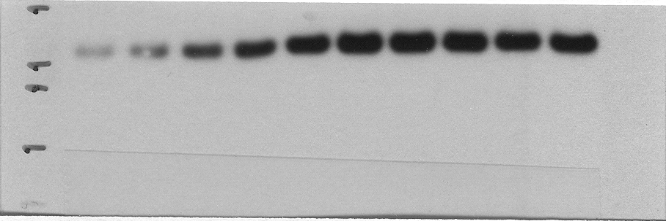
**

**Pfeiffer (DLBCL) - pS6 (Ser235/236)**

**
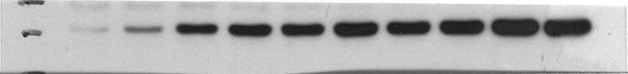
**

**Pfeiffer (DLBCL) - pp70S6K (Thr389)**

**
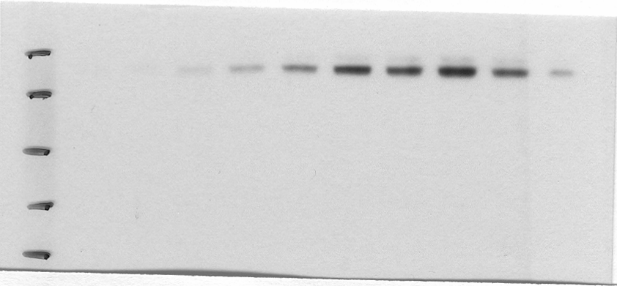
**

**Pfeiffer (DLBCL) - p4E-BP1 (Ser65)**

**
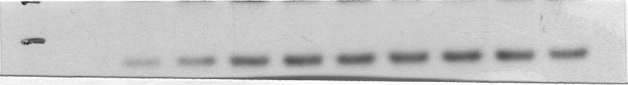
**

**KMS-12-PE (MM) - pS6 (Ser235/236)**

**
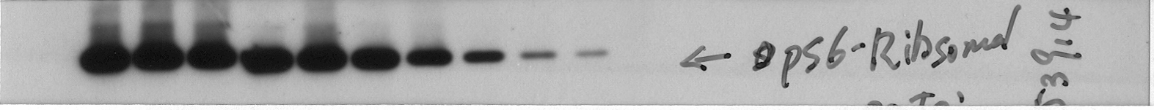
**

**KMS-12-PE (MM) - pp70S6K (Thr389)**

**
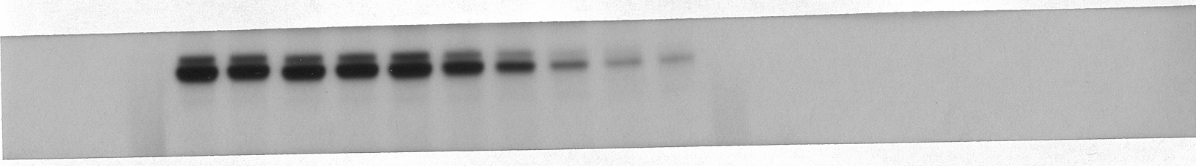
**

**KMS-12-PE (MM) - p4E-BP1 (Ser65)**

**
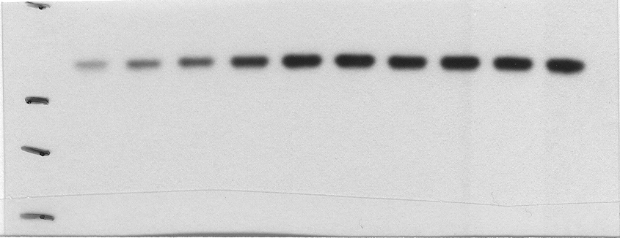
**

**KMS-12-BM (MM) - pS6 (Ser235/236)**

**
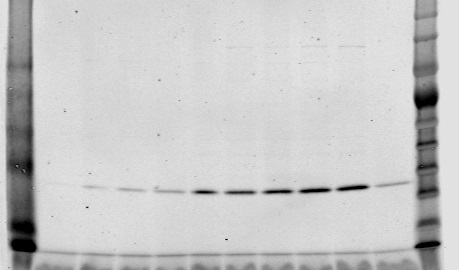
**

**KMS-12-BM (MM) - p4E-BP1 (Ser65)**

**
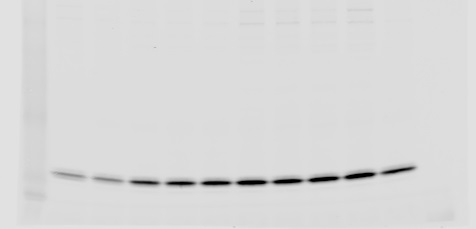
**

**Figure 2 Panel D**

**KG1a (AML) – PIM2**

**
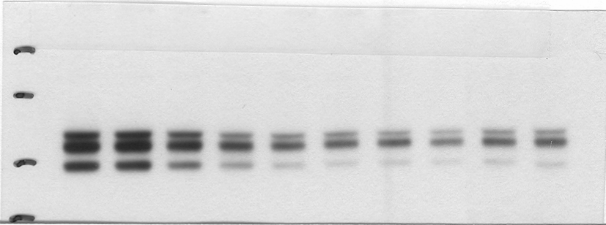
**

**Pfeiffer (DLBCL) – PIM2**

**
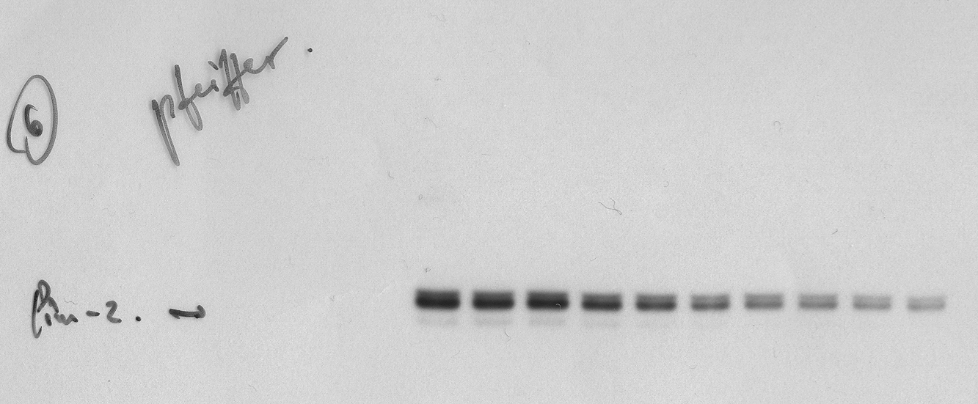
**

**KMS-12_PE (MM) – PIM2**

**
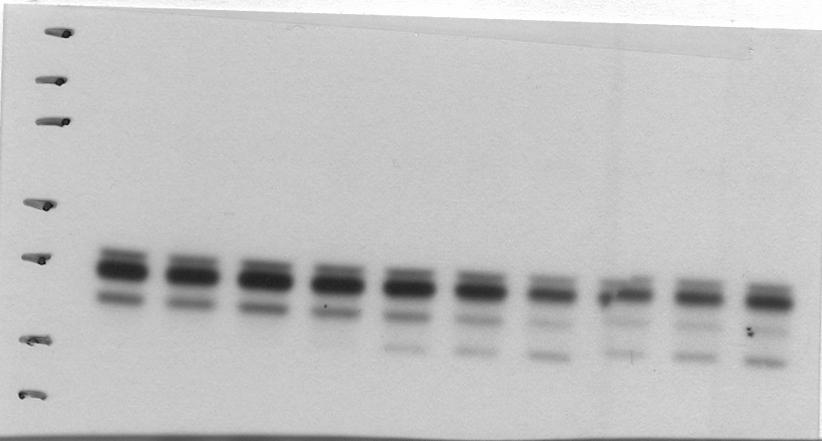
**

**Figure 3 Panel A**

**Samples A, B, and C**

**Figure 3 Panel A**

**Sample D**


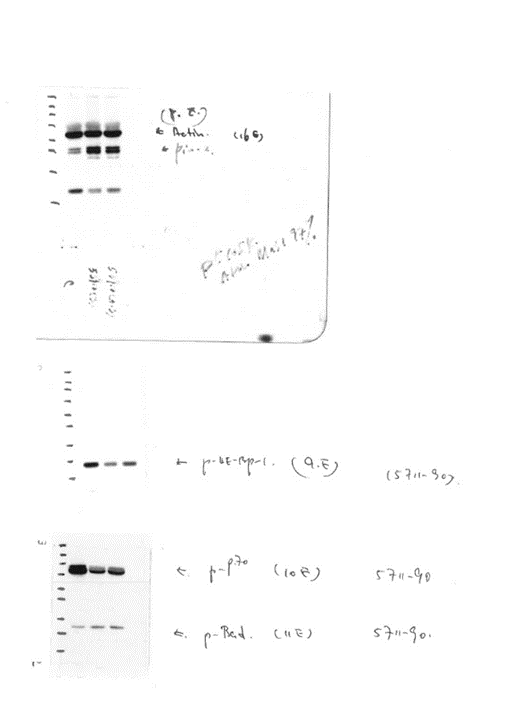


**Figure 3 Panel C**

**Patient 1**

**Patient 2**


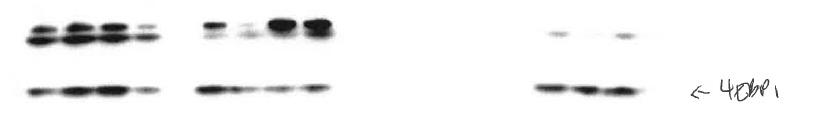

Supplement: S4 File — (DOCX) [file pone.0199108.s008.docx]
